# Gibberellin Promotes Sweetpotato Root Vascular Lignification and Reduces Storage-Root Formation

**DOI:** 10.3389/fpls.2019.01320

**Published:** 2019-11-15

**Authors:** Vikram Singh, Lidiya Sergeeva, Wilco Ligterink, Roni Aloni, Hanita Zemach, Adi Doron-Faigenboim, Jun Yang, Peng Zhang, Sara Shabtai, Nurit Firon

**Affiliations:** ^1^Department of Vegetable and Field Crops, Institute of Plant Sciences, Agricultural Research Organization, The Volcani Center, Rishon LeZion, Israel; ^2^Laboratory of Plant Physiology, Department of Plant Sciences, Wageningen University, Wageningen, Netherlands; ^3^School of Plant Sciences and Food Security, Tel Aviv University, Tel Aviv, Israel; ^4^Department of Fruit Tree Sciences, Institute of Plant Sciences, Agricultural Research Organization, The Volcani Center, Rishon LeZion, Israel; ^5^Chenshan Plant Science Research Center, Chinese Academy of Sciences, Shanghai Chenshan Botanical Garden, Shanghai, China; ^6^Institute of Plant Physiology & Ecology, SIBS, Chinese Academy of Sciences, Shanghai, China

**Keywords:** gene expression, gibberellin, lignin, root anatomy, storage-root, sweetpotato, xylem, yield

## Abstract

Sweetpotato yield depends on a change in the developmental fate of adventitious roots into storage-roots. The mechanisms underlying this developmental switch are still unclear. We examined the hypothesis claiming that regulation of root lignification determines storage-root formation. We show that application of the plant hormone gibberellin increased stem elongation and root gibberellin levels, while having inhibitory effects on root system parameters, decreasing lateral root number and length, and significantly reducing storage-root number and diameter. Furthermore, gibberellin enhanced root xylem development, caused increased lignin deposition, and, at the same time, decreased root starch accumulation. In accordance with these developmental effects, gibberellin application upregulated expression levels of sweetpotato orthologues of *Arabidopsis* vascular development regulators (*IbNA075*, *IbVND7*, and *IbSND2*) and of lignin biosynthesis genes (*IbPAL*, *IbC4H*, *Ib4CL*, *IbCCoAOMT*, and *IbCAD*), while downregulating starch biosynthesis genes (*IbAGPase* and *IbGBSS*) in the roots. Interestingly, gibberellin downregulated root expression levels of orthologues of the *Arabidopsis BREVIPEDICELLUS* transcription factor (*IbKN2* and *IbKN3*), regulator of meristem maintenance. The results substantiate our hypothesis and mark gibberellin as an important player in regulation of sweetpotato root development, suggesting that increased fiber formation and lignification inhibit storage-root formation and yield. Taken together, our findings provide insight into the mechanisms underlying sweetpotato storage-root formation and provide a valuable database of genes for further research.

## Highlights

Gibberellin affects sweetpotato yield, causing upregulation of root xylem development and lignin biosynthesis, together with downregulation of starch accumulation and inhibition of storage-root formation.

## Introduction

Sweetpotato (*Ipomoea batatas* (L.) Lam., family *Convolvulaceae*) is the sixth most important food crop globally, after wheat, rice, maize, potato, and cassava (CIP, 2017), with 112.8 million tons produced in 2017 (FAOSTAT, 2017). Sweetpotato is a rich source of carbohydrates, vitamins, dietary fiber, and micronutrients, and is of high importance for food security and for improving the nutrition status in Asia and especially in sub-Saharan Africa. Sweetpotato can grow under marginal conditions, thus providing a good solution for nutrition in developing countries (CIP, 2017).

The most important process in sweetpotato production is storage-root (SR) formation. Sweetpotato plants are propagated through stem-cuttings that produce adventitious roots (ARs) from primordia developed on stem nodes (Ma et al., 2015). Initially, these ARs develop into white lignified roots that support development of the sweetpotato plant. Most of these initial ARs can, however, change their developmental fate and be transformed into SRs (Villordon et al., 2009). This change involves accumulation of starch and is dependent on regulatory mechanisms that are not yet well characterized (Firon et al., 2013; Ravi et al., 2014). The first clear sign of the developmental transition into a SR is formation of primary and secondary cambial cells (anomalous cambium) encircling the AR primary and secondary xylem elements (Villordon et al., 2009). In SRs, cambial cells proliferation occurs, forming starch-accumulating parenchyma cells in the root vascular cylinder (Villordon et al., 2009). In roots that do not develop into SRs, intensive stele lignification is documented (Villordon et al., 2009), suggesting that stele lignification during the early phase of root development affects SR development (Togari, 1950). By performing transcriptional profiling of sweetpotato roots during SR initiation phase, we have previously suggested that down-regulation of lignin biosynthesis and up-regulation of starch biosynthesis are key events at the early stage of SR formation (Firon et al., 2013). A link between root system architecture (RSA) parameters and SR initiation and yield potential was suggested (Villordon et al., 2012; Villordon and Clark, 2014; Villordon et al., 2014). Adventitious roots that showed evidence for SR initiation had higher lateral root (LR) attributes such as LR number, length and surface area (Villordon et al., 2012). It was further suggested that in ARs with a prevalence of arrested/non-emerged LR primordia, the adjacent stellar tissue becomes lignified, rendering it incapable of undergoing swelling due to the lack of vascular and anomalous cambia development (Villordon and Clark, 2014). Thus, a relationship between RSA and the developmental fate of sweetpotato ARs was suggested.

Lignification is considered part of the plant’s developmental program, being coordinated with the production of cellulose, hemicellulose, and other polysaccharides during secondary wall formation (Bonawitz and Chapple, 2010 and references therein). In *Arabidopsis*, significant metabolic commitment to secondary wall deposition occurs during maturation of xylem vessels, xylary fiber cells, and interfascicular fiber cells in the developing inflorescence stem (Chaffey et al., 2002; Zhong et al., 2006). Such a link between lignification and xylem development was established in numerous systems (Kaneda et al., 2008; Geng et al., 2018). In cassava roots, similar to sweetpotato, the root system consists mainly of two root types, fibrous/lignified roots that transport water and nutrients, and SRs that accumulate starch. The fibrous roots produce xylem vessels and xylem fibers, and SRs produce mainly starch-storing xylem parenchyma cells (Yang et al., 2011). Recently, a correlation was established in cassava between expression of xylem differentiation- and lignin-biosynthesis-regulators, and fibrous root development (Siebers et al., 2017).

Lignification is governed mainly by genes involved in lignin biosynthesis and by master regulators of vascular development, regulating xylem, and xylem fiber cell differentiation, including members of the vascular-related NAC domain (VND) (Ohashi-Ito et al., 2010) and NAC secondary-wall thickening promoting factor/secondary-wall-associated NAC domain protein (NST/SND) (Zhong et al., 2006; Zhong et al., 2007). Most information on these genes is available from studies in *Arabidopsis* (Yamaguchi et al., 2008; Hussey et al., 2011) and wood formation (Hellmann et al., 2018). These upstream regulatory NAC domain transcription factors act as either activators or repressors of lignin biosynthesis (Taylor-Teeples et al., 2015). Among these, the positive regulators VND5, 6, and 7 are master switches of xylem cell differentiation, regulating protoxylem, and metaxylem differentiation, and secondary wall biosynthesis (Kubo et al., 2005; Yamaguchi et al., 2008; Zhou et al., 2014). SND1/NST1 and SND2 are involved in secondary cell wall formation in xylem vessels and xylem fiber differentiation (Zhong et al., 2006; Mitsuda et al., 2007; Hussey et al., 2011). The NAC domain repressor, VND-INTERACTING 2 (VNI2) negatively regulates xylem vessel formation/differentiation and represses VND7-induced expression of vessel-specific genes (Yamaguchi et al., 2010). Another NAC domain repressor, XYLEM NAC DOMAIN 1 (XND1) was also shown to reduce xylem vessel differentiation and lignin accumulation (Zhao et al., 2008). Recently, *VND* and *NST* genes were suggested as potential regulators of xylem specification in cassava roots (Siebers et al., 2017). In sweetpotato, downregulation of various NAC domain transcription factors was reported during SR formation (McGregor, 2006).

Lignin biosynthesis (being the linking of monolignol units) depends on the monolignol biosynthesis pathway, starting with deamination of phenylalanine by phenylalanine ammonia-lyase (PAL; the main enzyme of the phenylpropanoid pathway) (Boerjan et al., 2003). This is followed by a series of reactions, involving the following enzymes: cinnamate 4-hydroxylase (C4H), 4-coumarate:CoA ligase (4CL), *p*-hydroxycinnamoyl-CoA: quinate shikimate *p*-hydroxycinnamoyltransferase (HCT), caffeoyl-CoA *O*-methyltransferase (CCoAOMT), and cinnamyl alcohol dehydrogenase (CAD) (Raes et al., 2003). Downregulation of genes involved in lignin biosynthesis, including *PAL*, *C4H*, *4CL*, *HCT, CCoAOMT*, and *CAD* was reported during sweetpotato SR formation (Firon et al., 2013; Tanaka, 2016). Moreover, up-regulation of key enzymes of the phenylpropanoid biosynthesis pathway in sweetpotato roots, by overexpressing the maize leaf color gene, was found to correlate with higher lignification, lower starch accumulation, and lower SR yield (Wang et al., 2016).

Recently, it was demonstrated that the plant hormone gibberellin (GA) is involved in root growth, secondary xylem development and lignin accumulation in carrot (Wang et al., 2015a; Wang et al., 2017). Exogenous application of GA_3_ was shown in *Betula platyphylla* to induce xylem development and expression of secondary wall biosynthesis genes (Guo et al., 2015). In Aspen, it was suggested that GA has a role in regulating early stages of xylem differentiation during wood formation (Israelsson et al., 2005). Gibberellin is known to regulate diverse plant developmental processes throughout the life cycle, like stem elongation and seed germination (Gupta and Chakrabarty, 2013). It was shown to affect xylem formation and plant lignification in various systems, causing upregulation in expression of lignin biosynthesis genes (Biemelt et al., 2004). Gibberellins exist as bioactive (GA_1_, GA_3_, GA_4,_ and GA_7_) and inactive forms (intermediates, precursors, and catabolites), the level of bioactive GAs being maintained by feedback and feedforward regulation of GA metabolism/biosynthesis and deactivation/degradation pathways (Hedden and Phillips, 2000; Olszewski et al., 2002). Gibberellin biosynthesis is regulated by *ent*-*kaurene oxidase (KO*), *GA 20-oxidase* (*GA20ox*), and *GA 3-oxidase* (*GA3ox*) genes and degradation is governed by a family of GA 2-oxidase isoforms (*GA2ox*) (Hedden and Phillips, 2000). The GA signaling pathway starts by binding of GA to its receptor, GA Insensitive Dwarf1 (GID1), which enhances an interaction between GID1 and DELLA that causes DELLA degradation and de-repression of the pathway (Sun, 2011). Another important player in GA regulation is gibberellic acid insensitive (GAI), being a repressor of GA responses (Peng et al., 1997).

Storage-root initiation involves proliferation of vascular cambial cells and starch accumulation (Firon et al., 2013; Ravi et al., 2014). In this context, it is of interest to highlight class I knotted 1-like (*KNOX*) genes, pointed out previously in different plant systems (*Arabidopsis*, poplar, and sweetpotato) as regulators of plant meristems, including cambial cells (Schrader et al., 2004; Scofield and Murray, 2006; Tanaka et al., 2008). Several *KNOX* gene sequences were previously identified by us to be upregulated in initiating sweetpotato SRs (Firon et al., 2013), including two *Arabidopsis BREVIPEDICELLUS* (*BP*) orthologues. Interestingly, it was found that BP can regulate lignin biosynthesis. Overexpression of *BP* was found to cause decreased lignification and *bp* mutants exhibited elevated lignin levels (Mele et al., 2003). The possibility of binding of BP to lignin biosynthesis genes promoters was demonstrated (Mele et al., 2003). Another link was demonstrated between the *BP* gene and GA, showing that BP may negatively regulate GA (Bolduc and Hake, 2009). In tobacco, overexpression of a KNOTTED-type protein caused decreased expression of a GA biosynthesis gene (Tanaka-Ueguchi et al., 1998). Hay et al. (2002) suggested that “repression of GA activity by KNOX transcription factors is a key component of meristem function.” During sweetpotato SR formation, vascular cambial cells develop into starch-accumulating parenchyma cells, exhibiting upregulated expression of genes involved in starch biosynthesis (Firon et al., 2013). Such genes include phosphoglucomutase (*PGM*), ADP glucose pyrophosphorylase (*AGPase*), granule-bound starch synthase (*GBSS*), and starch phosphorylase (*SP*) (Geigenberger, 2011).

In the present study, in order to better characterize the complex events that take place during the change of the sweetpotato AR into a SR, we looked into the effect of exogenous application of GA_3_ on root development and root lignin accumulation, together with vascular system- and xylem-development, starch accumulation, transcript levels of genes that regulate all these processes as well as the number of SRs formed. The results show that GA_3_ application influenced sweetpotato plant development by increasing stem elongation, caused a significant increase in levels of root bioactive GAs, decreased the number of ARs as well as the number and length of LRs, and reduced significantly the number of SRs. In addition, application of GA_3_ influenced root xylem development, caused increased lignin deposition, and decreased starch accumulation. The effect of GA_3_ application on starch accumulation was apparent as early as two weeks after planting. In accordance with these developmental effects, GA_3_ application was found to cause upregulated expression levels of sweetpotato orthologues of vascular development regulators (*IbNA075, IbVND7*, and *IbSND2*) and of lignin biosynthesis genes (*IbPAL*, *IbC4H*, *Ib4CL*, *IbCCoAOMT*, and *IbCAD*) and downregulation in expression levels of *KNOX* (*IbKN2* and *IbKN3*) and starch biosynthesis (*IbAGPase* and *IbGBSS*) genes. These results point to GA, and potentially *KNOX* genes, as important regulators of root xylem development and lignification, on one hand, and formation of starch-accumulating cells, on the other hand, regulating SR formation and yield.

## Materials and Methods

### Plant Material, Growth Conditions, GA_3_, and Paclobutrazol Treatments

Virus-tested sweetpotato stem cuttings were obtained from plants of the “*Georgia Jet*” cultivar, grown commercially in the Hasharon region, Israel. Stem cuttings with three nodes (nodes number 10 to 12 from the plant apex) were used for the study as described previously (Ma et al., 2015). Before planting, leaves of node 12 were removed and cuttings (node 12) were dipped separately into either water (“control”), a solution of gibberellic acid 3 (“GA_3_”; 50 ppm diluted in water before use, following calibration of GA concentrations in the range of 10 to 1000 ppm, for getting an obvious, but not drastic, effect on plant development) or an inhibitor of GA synthesis, paclobutrazol (“PB”; 5 ppm in water, following calibration in the range of 1 to 20 ppm) (Wang et al., 1986), for 18 h in room temperature.

Thereafter, cuttings were planted into PVC pots (10 cm diameter, 30 cm tall) prefilled with washed sand, by putting one node (node 12) in the soil. Subsequently, pots were transferred to a greenhouse at the Volcani Center, Rishon LeZion, Israel. The experiment was conducted during April 2017. Greenhouse temperature was maintained at 25/20°C ± 3 day/night temperatures under natural conditions, with no supplemental light. Minimum 16 cuttings were planted for each treatment group and each sampling time. A total of three treatments were used, as detailed below, and the plants were randomly arranged in one greenhouse. Plants were irrigated with 100 mL of either water (half field capacity; “control” treatment), an aqueous solution of 50 ppm GA_3_, or 5 ppm PB (“GA” and “PB” treatments, respectively) on every third day, until two weeks (2W) after planting. Thereafter plants were irrigated with a low N fertilizer solution (100 mg/L of 20: 20: 20 N: P: K; Haifa Chemicals, Israel), twice a week, until the end of the experiment.

### Plant Development Analyses

For following the effect of GA_3_ and PB treatments on plant development, lateral stem length (originated from node 10) was measured at 3 weeks (3W), 4W, and 5W after planting, using 16 plants (biological replicates) (**Supplementary Figure S1**). For looking into the effect of GA_3_ and PB on sweetpotato root development, the whole root system (originated from node 12) was sampled at 1W, 2W, and 5W after planting from each of the three treatment groups, using four plants per treatment out of the 16 planted plants (**Supplementary Figure S1**). These time points were chosen to represent (i) the phase in root development during which a developmental decision will be made toward becoming either a storage-root or a lignified non-storage-root (the first 2 weeks after planting; (Villordon et al., 2009) and (ii) the time when SR formation is observed (5W).

### Root Image Acquisition and Root System Architecture Analysis

The whole root system was sampled at 1W, 2W, and 5W after planting as indicated above and maintained in 30% ethanol at 4°C until analyzed. For analysis, roots were floated in a water-filled tray, and scanned by an optical scanner (Epson Expression 1600, Japan). Images were analyzed using the WinRHIZO software version 5b (Regent Instruments Inc., Quebec, Canada), and by ImageJ software [ImageJ 1.51a, NIH, USA, (Schneider et al., 2012)]. Measured root parameters included: average root system fresh weight (FW; using 16 plants), root system dry weight (DW; analyzed after 72 h incubation in an oven at 60°C, using four plants), number of AR per plant (counted manually), AR cumulative length per plant (calculated by ImageJ software from scanned images of WinRHIZO), average number of LR per plant (calculated by, “total tips count obtained from WinRHIZO analysis” minus “AR number”), LR cumulative length per plant (calculated by “total root length obtained from WinRHIZO analysis” minus “AR length”), LR density (LR number divided by AR length), and root surface area (derived from WinRHIZO analysis).The five parameters, AR and LR numbers, AR and LR length, and LR density were measured at 1W and 2W after planting using four biological replicates (plants). Average root system FW and DW, root volume, and root surface area per plant were recorded at 5W after planting using four biological replicates (plants). The measured values included all root types (AR, LR, and SR) present at the time of sampling.

### Storage-Root Parameters

Storage-root number per plant was evaluated at 5W after planting using 16 plants per treatment (control, 50 ppm GA_3_ and 5 ppm PB). Analyzed SR parameters included average diameter (sampled from four plants) and average FW (sampled from 16 plants). It should be noted that the same 12 plants, per each treatment and sampling date, were used for looking into root anatomy, quantifying root endogenous GA levels and starch levels, and for gene expression analysis (RNA extraction) as detailed below and described in **Supplementary Figure S1**. For root anatomy, samples were fixed in FAA solution (1 L) containing; 100 mL of 35% formaldehyde, 50 mL of glacial acetic acid, 520 mL of 96% ethanol, and 330 mL of dH_2_O. Sampled roots for endogenous GA, starch, and gene expression analyses were plunged immediately into liquid nitrogen and stored at -80°C until use.

### Quantification of Endogenous Gibberellins

Endogenous GAs levels were determined in sweetpotato ARs, derived from 12 plants which were sampled at 2W after planting, according to the established protocol (Kolachevskaya et al., 2017). Fine-homogenized lyophilized root samples (10 mg) were extracted with 1 mL of 80% acetonitrile with 5% formic acid and deuterium-labeled GA internal standards [^2^H_2_]GA_1_, [^2^H_2_]GA_3_, [^2^H_2_]GA_4_, [^2^H_2_]GA_7_, and [^2^H_2_]GA_20_ (10 pmol of each compound). In the first step, samples were sonicated (5 min), rotated for 12 h (at 4°C), and centrifuged for 10 min at 17,000 rpm (at 4°C). Supernatant was collected and kept at -20°C. The remained pellet was re-extracted with 1 mL of the extraction solvent without adding internal standards, sonicated, rotated for 1 h at 4°C, and centrifuged for 10 min at 17,000 rpm (at 4°C). Combined supernatants were dried in a Speed-Vac concentrator (Savant SC210A, Thermo, US), reconstituted by 5% of NH_4_OH (v/v) and loaded on Oasis® MAX column (6 cc/150 mg, Waters Co., Milford, MA, USA). Before loading samples, the column was activated with 100% MeOH, washed with mQ water, and equilibrated with 5% of NH_4_OH. Compounds were eluted with 0.2 M formic acid in acetonitrile and the eluate was dried *in vacuo*. Thereafter, samples were reconstituted in 60 µL of initial mobile phase 10 mM formic acid: MeOH (80: 20, v/v), filtered through micro spin filters 0.2 µm (Thermo Fisher Scientific, USA) and injected onto reversed-phase UPLC CSH C18 column (100 x 2.1 mm, 1.7 µm) of Acquity UPLC^TM^ I-Class Core System (Waters, USA). GAs were separated isocratically at 80% of 10 mM formic acid/water (A, v/v) and 20% of methanol (B) at a flow rate of 0.35 mL/min for 1 min, followed by consecutive linear gradient to 70 and 90% of B over 9 min. Column was thermostated at 50°C. The effluent was introduced into electrospray ion source of Xevo® TQ-S triple quadrupole mass spectrometer (Waters MS Technologies, Manchester, UK), having the capillary voltage of 3 kV. The source block/desolvation temperature was adjusted to 120°C/550°C, and desolvation gas flow rate was maintained by 650 L h^-1^. Compounds were quantified by multiple ion monitoring mode (MRM) as described (Kolachevskaya et al., 2017). Data were processed using MassLynx^TM^ software with TargetLynx^TM^ program (version 4.1, Waters, USA).

### Analysis of Starch Levels

Starch level was determined in ARs (sampled at 2W and 5W after planting) and in SRs (sampled at 5W) following the protocol of (MacRae (1971) with minor modifications. Powdered samples (250 mg), obtained following grinding in liquid N_2_, were treated with 6 ml of 80% ethanol and incubated at 70°C for 45 min. Following incubation, ethanol was discarded, fresh 80% ethanol was added and samples were incubated, as described above, this step being repeated twice. Finally, samples containing fresh 6 ml of 80% ethanol were incubated at 70°C for 20 min. After incubation, ethanol was discarded and tubes containing ethanol-insoluble sugar (starch) were kept in an oven at 60°C for drying overnight. Thereafter, 6 ml of dH_2_O was added to the dried samples and were autoclaved at 121°C for 60 min. Subsequently, samples were cooled down to room temperature and 4 ml of working solution, containing 0.5 ml acetate buffer (2M, pH 4.8), 2.5 ml dH_2_O, and 1 ml of amyloglucosidase (10 mg/ml), was added to each sample. Tubes were covered with aluminum foil and kept for overnight shaking at 55°C in a water bath. Following overnight incubation, 250 µl of sample solution was transferred into a 1.5 ml eppendorf tube, adding 250 µl of dH_2_O and 500 µl of Sumner reagent. Tubes were covered with aluminum foil and boiled in hot water for 5 min to follow the change in color from yellow to orange. Tubes were allowed to cool down at room temperature and measurements were done at 550 nm in a spectrophotometer (Pharmacia Biotech, USA). A glucose solution (0.04%) was used for generating a standard curve.

### Histochemical Analysis and Autofluorescence Imaging

Roots were sampled from six to eight plants from each treatment group (“control,” 50 ppm “GA,” and 5 ppm “PB”) at 1W, 2W, and 5W after planting (5W samples were divided into lignified non-SR/AR and SR). Samples stored in FAA solution (as mentioned above) were dehydrated using ethanol dilution series, followed by embedding in paraffin wax (Ruzin, 1999). Sectioning was done by a microtome (Leica RM2245, Leica Biosystems, Nussloch, Germany) and 15 µm thick sections were prepared. Following sectioning, root sections were deparaffinized in a histoclear solution, rehydrated with ethanol dilution series, and used further for histochemical staining and autofluorescence imaging. Histochemical staining was done by either, safranin-fast green or Phloroglucinol-HCl (Ph-HCl or Weisner) staining to observe the root vascular system and lignin deposition (phloroglucinol is a good indicator of general cinnamaldehydes), respectively. For safranin-fast green staining, deparaffinized samples were stained for 2 h in safranin-O (1%) with counter staining for 10 s in fast green (0.5%). Phloroglucinol-HCl staining was done according to an established protocol by Mitra and Loque (2014), where deparaffinized samples were stained with a freshly prepared Ph-HCl stain and observed immediately under the microscope (Ph-HCl causes sample deterioration). Microscopic examination of sections was done by a light microscope (Leica, Germany) and images were digitally captured by a Nikon DS-Fi1 digital camera. Autofluorescence imaging of unstained, deparaffinized sections was done by Confocal microscopy (Donaldson and Knox, 2012). Autofluorescence was detected under UV excitation at 365 nm with a PMT detector in range of 415–490 nm. Using UV is a simple method to visualize lignin and other aromatic molecules. All microscopic observations and image acquisitions were performed by a Leica SP8 laser scanning microscope (Leica, Wetzlar, Germany), equipped with a solid-state laser with 405 nm light, HC PL APO CS 10x/0.40 objective (Leica, Wetzlar, Germany), and Leica Application Suite X software (LASX, Leica, Wetzlar, Germany).

Root vascular parameters were determined from images captured after histochemical analysis. Total xylem vessel number was calculated by counting the number of protoxylem, metaxylem and secondary xylem elements in root sections. Moreover, area covered by xylem elements (vessels and fibers) and total root area were calculated using the imagej software (Imagej 1.51a, NIH, USA, (Schneider et al., 2012), and percent root area occupied by xylem vessels (protoxylem, metaxylem, and secondary xylem) and xylem fibers was determined.

### RNA Extraction and Gene Expression Analysis

Total RNA was extracted from sweetpotato ARs at 1W, 2W, and 5W after planting (5W sampled roots included both, ARs and SRs), using RNeasy Plant Mini Kit (Qiagen, Germany). The integrity and quantity of RNA was examined by gel-electrophoresis and nanodrop (ND 1000, Thermo Scientific, USA), respectively. DNA contamination from RNA samples was removed using TURBO DNA-*free* ^TM^ kit (Ambion, Life Technology, USA). The cDNA was prepared using the Verso cDNA Synthesis Kit according to the manufacturer’s protocol (Thermo Scientific, *Lithuania*), and was used for further analyses.

Gene expression analysis was performed by quantitative reverse transcriptase-PCR (qRT-PCR) using a 10 µL reaction volume containing cDNA, forward and reverse primers, and ABsolute Blue qPCR SYBR Green ROX Mix (Thermo Scientific, *Lithuania*). Reactions were performed in a *Rotor Gene 6000* Real-Time PCR System (Corbett Life Science, Australia) using 40 cycles of 10 s at 95°C, 15 s at 60°C, and 20 s at 72°C. Results were analyzed by Rotor gene software, and relative expression levels of targeted genes were calculated by the 2^-ΔCt^ method. The selection of a reference gene was done according to Park et al. (2012). Total 10 candidate reference genes, including beta actin, ribosomal protein L, glyceraldehyde-3- phosphate dehydrogenase, cyclophilin, a-tubulin, ADP-ribosylation factor, histone H2B, ubiquitin extension protein, cytochrome c oxidase subunit Vc, and phospholipase D1a were tested. Among these, phospholipase D1a was found to be most suitable, having stable gene expression, and was used for data normalization in our experiments.

Primers were designed using Primer3Plus^1^ and are listed in **Supplementary Table S1**.

### Sequence and Statistical Analyses

BLAST and tBLASTx were used to compare sequences of candidate sweetpotato contigs/genes of GA biosynthesis and signaling, lignin biosynthesis, and upstream regulators, class I knotted 1-like and starch metabolism, derived from our published database (Firon et al., 2013) with *Arabidopsis* ^2^ and NCBI^3^ databases. For NAC-domain-containing sequences, in order to identify potential up-regulators of lignin and xylem development, homology against PlantTFDB database^4^ was performed. Selected sweetpotato NAC genes were further investigated by motif search^5^, and phylogenetic (using MEGA with neighbor-joining method and 1000 bootstrap replicates) analyses for their similarly to *Arabidopsis* genes, in order to identify potential orthologues of *NAC075*, *VND*, and *SND* genes (**Supplementary Figure S2**).

Data statistical analyses were performed using student’s t-test at *P* ≤ 0.05, using JMP 5.0.1a statistical software (SAS Institute Inc., NC, United States).

## Results

### Exogenous Application of GA_3_ Influences Sweetpotato Stem Growth and Root System Architecture

**Figure 1:**
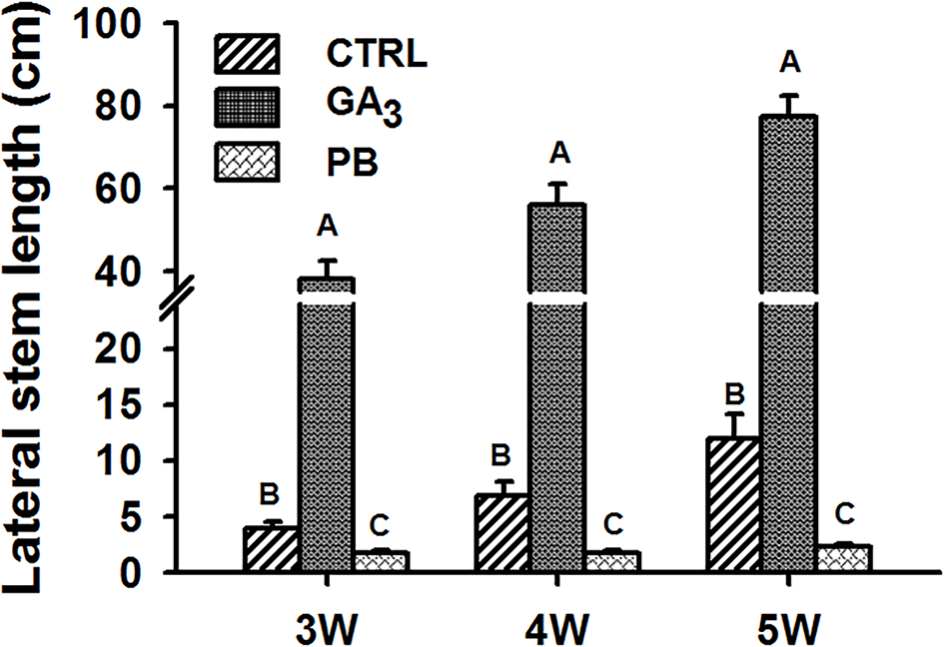
Effect of GA_3_ application on sweetpotato “*Georgia Jet*” stem growth. Lateral stem length was recorded at three, four, and five weeks (3W, 4W, and 5W, respectively) after planting. Bars represent mean of 16 independent biological replicates (plants) ± SE. Significance analysis was performed by using student’s t-test (*P ≤ 0.05)*, where unlike letters represent significant differences between treatments within a sampling group. CTRL, Control (treated with water); GA_3_, application of 50 ppm gibberellic acid 3 for two weeks; PB, application of 5 ppm paclobutrazol for two weeks.

The effect of exogenous application of GA_3_ and of the GA biosynthesis inhibitor PB was tested on stem growth and on root characteristics. The experimental setup and conditions used are detailed in **Supplementary Figure S1**. The lateral stem length measured at 3W, 4W, and 5W after planting was found to change significantly by GA_3_ and PB treatments as compared to control (**Figure 1**). The stem was 9.7-, 8.1-, and 6.5-fold longer following GA_3_ treatment, while PB treatment caused stem shortening compared to control by 2.3-, 4.0-, and 5.3-fold, at 3W, 4W, and 5W, respectively. Thus, GA_3_ and PB showed antagonistic effects causing elongated or stunted stem growth, respectively, throughout sweetpotato plant development.

**Figure 2:**
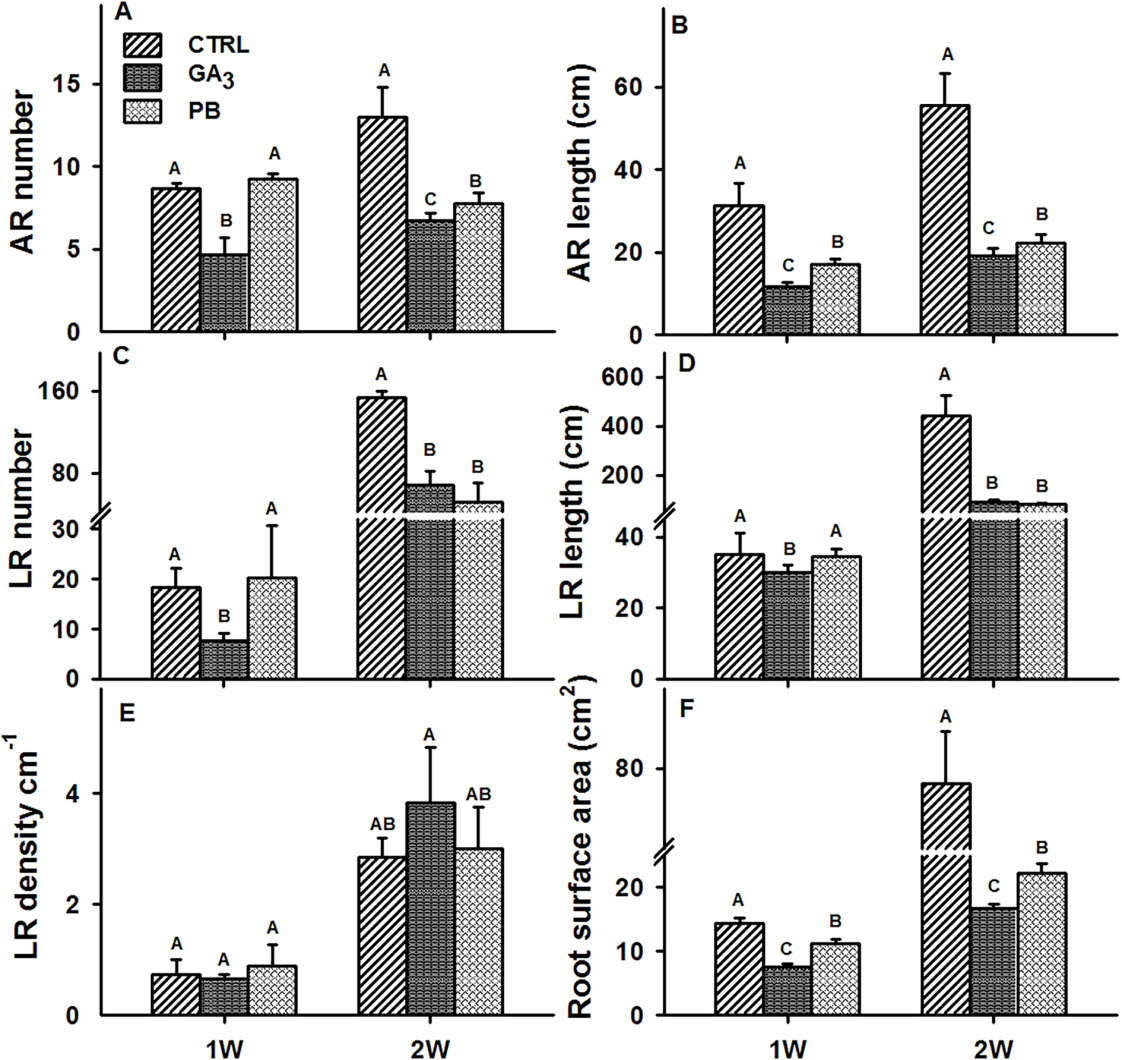
Effect of GA_3_ application on sweetpotato “*Georgia Jet*” root system architecture parameters. The parameters included adventitious root (AR) number per plant **(A)**, AR cumulative length per plant **(B)**, lateral root (LR) number per plant **(C)**, LR cumulative length per plant **(D)**, LR density per AR **(E)**, and root surface area per plant **(F)**, determined at one and two weeks (1W and 2W, respectively) after planting. Bars represent mean of four independent biological replicates (plants) ± SE. Significance analysis was performed by using student’s t-test (*P ≤ 0.05)*, where unlike letters represent significant differences between treatments within a sampling group. CTRL, Control (treated with water); GA_3_, application of 50 ppm gibberellic acid 3 for two weeks; PB, application of 5 ppm paclobutrazol for two weeks.

We have previously demonstrated that the initial stages of AR development are critical in determining SR set in sweetpotato (Villordon et al., 2009). For this reason, the effect of exogenous application of GA_3_ and PB on root characteristics was studied during the first two weeks after planting. During this period, stem cuttings were treated with the hormone or inhibitor solution as described in *Materials and Methods*. The results of root system FW and DW at 1W and 2W after planting are presented in **Supplementary Figure S3**, showing a significant reduction in both parameters by both GA_3_ and PB at 2W (2.0- and 3.4-fold as well as 1.7- and 2.1-fold, for GA_3_ and PB respectively). Moreover, significant reduction in AR number, AR cumulative length, LR number, and LR cumulative length per plant was observed at 2W after planting following both, GA_3_ and PB treatments (**Figures 2A, B, C, and D**, respectively). For example, 1.9- and 2.9- as well as 1.7- and 2.5-fold reduction in AR number and length was observed at 2W, with GA_3_ and PB treatments, respectively (**Figures 2A, B**). While, 2.3- and 4.9- as well as 3.0- and 5.5-fold reduction in LR number and LR cumulative length, was detected following GA_3_ and PB treatments, respectively (**Figures 2C, D**). Adventitious root and LR number was reduced already at 1W after planting by GA_3_ and not by PB (**Figures 2A, C**, respectively). Lateral root density increased more than 3-fold at 2W as compared to 1W, under all tested conditions, and no significant differences were apparent following GA_3_ or PB applications (**Figure 2E**). As for root surface area, a significant decrease (more than 3-fold) was recorded for both GA_3_ and PB treatments at 2W after planting (**Figure 2F**).

### Exogenous Application of GA_3_ Affects Storage-Root Formation and Characteristics

**Figure 3:**
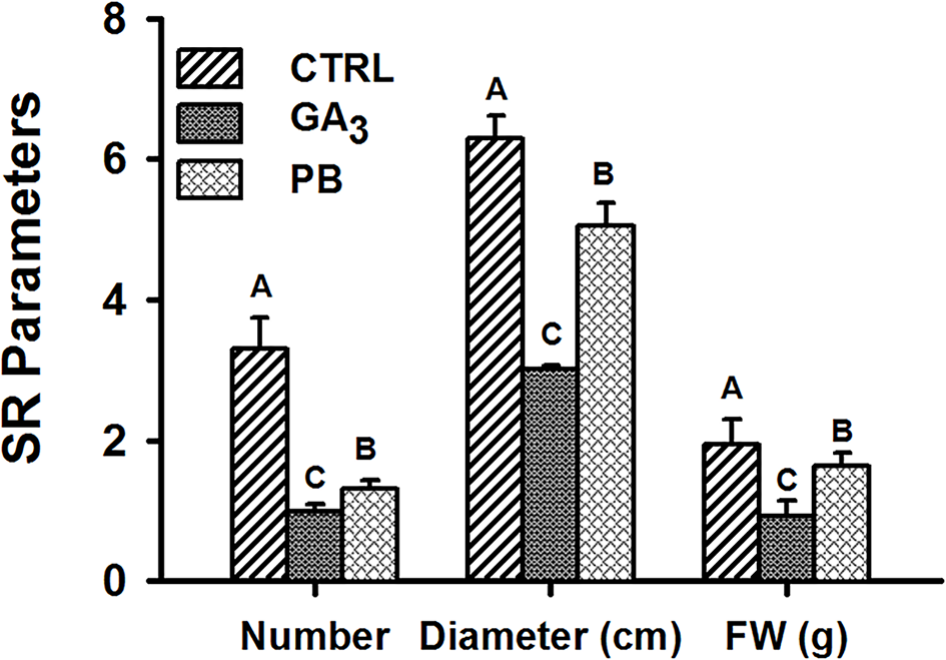
Effect of GA_3_ application on sweetpotato “*Georgia Jet*” storage-root characteristics. Storage-root (SR) number, average diameter and fresh weight (FW) per plant were recorded at five weeks (5W) after planting. Bars represent mean of 16 (for SR number and fresh weight; FW) or four (for SR diameter) independent biological replicates (plants) ± SE. Significance analysis was performed by using student’s t-test (*P ≤ 0.05*), where unlike letters represent significant differences between treatments within a sampling group. CTRL, Control (treated with water); GA_3_, application of 50 ppm gibberellic acid 3 for two weeks; PB, application of 5 ppm paclobutrazol for two weeks.

Root system architecture parameters, including root system FW, DW, volume, and surface area, were further recorded at 5W after planting, when SR formation was already detected. The results show significant reduction by both GA_3_ and PB treatments in all tested parameters (**Supplementary Figure S4**). Both GA_3_ and PB treatment affected SR formation as well as SR average diameter and weight, with GA_3_ causing a more pronounced effect. The reduction was apparent in SR number (3.3- and 2.5-fold), SR diameter (2.1- and 1.2-fold), and SR FW (2.1- and 1.2-fold), in response to GA_3_ and PB application, respectively (**Figure 3**).

### Changes in Endogenous GA Levels in the Sweetpotato Root System Following Exogenous GA_3_ Treatment

**Figure 4:**
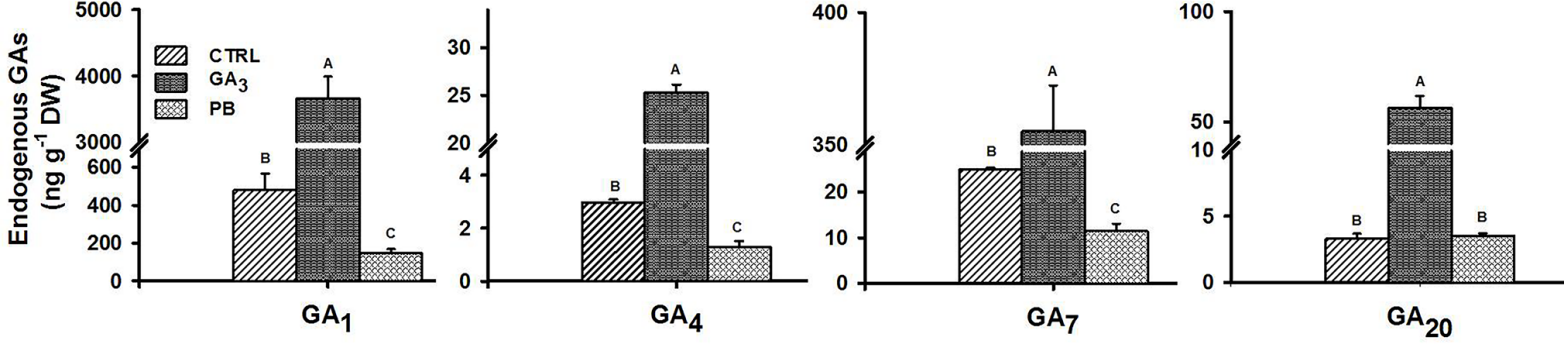
Effect of GA_3_ application on endogenous GAs levels in sweetpotato “*Georgia Jet*” roots. Levels of bioactive (GA_1_, GA_4,_ and GA_7_) and inactive (GA_20_) forms of GA, measured at two weeks (2W) after planting, are presented. Bars represent mean of three independent biological replicates (each consisting of roots pooled from four individual plants) ± SE. Significance analysis was performed by using student’s t-test (*P ≤ 0.05*), where unlike letters represent significant differences between treatments within a sampling group. CTRL, Control (treated with water); GA_3_, application of 50 ppm gibberellic acid 3 for two weeks; PB, application of 5 ppm paclobutrazol for two weeks. The content of GA_3_ (active form) was relatively high, reaching values 10,000, 70,000, and 4,000 ng g^-1^ DW for CTRL, GA_3,_ and PB treatment groups, respectively.

In order to examine the effect of exogenous GA_3_ or PB application on endogenous levels of GAs in sweetpotato roots, the 2W root samples from control and treated (GA_3_ or PB) plants were collected to quantify changes in GA levels, and results for major bioactive as well as inactive GAs, are presented in **Figure 4**. GA_3_ treatment caused a significant elevation (more than 7.0-fold) in the levels of all tested bioactive GAs (GA_1_, GA_3_, GA_4_, and GA_7_) and 17.2-fold for the measured inactive form GA_20_ (direct precursor of GA_1_ and precursor of GA_3_). Exogenous application of PB, caused the opposite effect, reducing the levels of all tested bioactive GAs by more than 2-fold. The content of GA_3_ was relatively high in all root samples, reaching values of 10,000; 70,000; and 4,000 ng g^-1^ DW in control, GA_3_- and PB-treated roots, respectively.

### Exogenous Application of GA_3_ Influences Sweetpotato Root Xylem Development and Lignification

**Figure 5:**
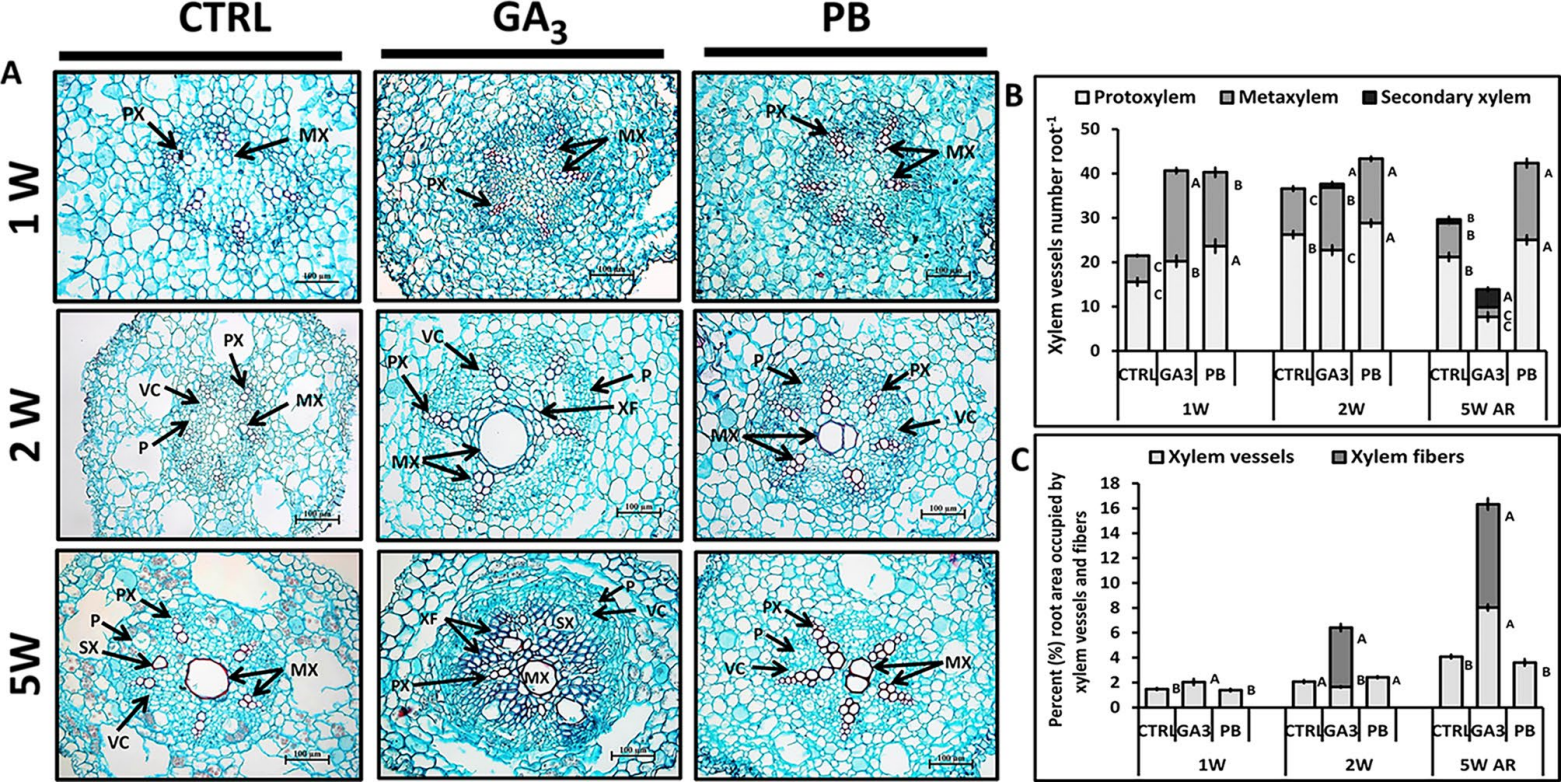
Effect of GA_3_ application on sweetpotato “*Georgia Jet*” root anatomy and xylem development. **(A)** Representative cross-sections of adventitious roots (AR) sampled at one, two, and five weeks (1W, 2W, and 5W, respectively) after planting. Sections were stained with safranin and fast green, and represent six to eight roots sampled from individual plants. Some of the vessels are lignified and stained red. **(B)** Xylem vessels number per AR, including protoxylem, metaxylem and secondary xylem sampled at 1W, 2W, and 5W. **(C)** Percent (%) root area occupied by xylem vessels (protoxylem, metaxylem and secondary xylem) and xylem fibers. Bars **(B**, **C)** represent mean of six to eight independent biological replicates (plants) ± SE. Significance analysis was performed by using student’s t-test (*P ≤ 0.05*), where unlike letters represent significant differences between treatments within a sampling group. CTRL, Control (treated with water); GA_3_, application of 50 ppm gibberellic acid 3 for two weeks; PB, application of 5 ppm paclobutrazol for two weeks. PX, protoxylem; MX, metaxylem; SX, secondary xylem; XF, xylem fibers; VC, vascular cambium; P, phloem. Scale bar = 100 µm.

In view of previous studies in other systems showing that GA_3_ promotes xylem development and vascular lignification (Guo et al., 2015; Wang et al., 2017), it was interesting to study its effect on sweetpotato root anatomy and on SR characteristics. Roots were thus harvested at the phase of SR initiation (1W and 2W) and at the time when SRs were already formed (5W) and analyzed for AR anatomical structure and xylem development. Analyses of the 5W samples were done using two root types; SR as well as AR that did not develop into a SR (sampled from the same plants). Cross-sections of ARs at 1W and 2W after planting show that root anatomy was influenced by both GA_3_ and PB treatments, exhibiting higher number of xylem vessels (**Figure 5A**). At this early phase of root development most xylem vessels were primary xylem (protoxylem and metaxylem) and a significant increase in protoxylem and metaxylem number at 1W, by both GA_3_ and PB treatments, was observed (**Figure 5B**). At 2W after planting, both GA_3_- and PB-treated roots exhibited significantly higher number of metaxylem cells compared to control, while the highest number of protoxylem cells was recorded following PB treatment (**Figure 5B**). GA_3_ treatment was found to promote secondary xylem formation, as evident in 2W and 5W cross-sections of ARs (**Figure 5B**). At 5W, those ARs that did not develop into SRs exhibited reduced number of protoxylem and metaxylem vessels and elevated number of secondary xylem vessels, following GA_3_ treatment as compared to control (**Figure 5B**). The 5W, GA_3_-treated AR sections exhibited formation of large secondary xylem vessels and a high number of xylem fibers (**Figure 5A**), manifested by the relative large area of the root section occupied by xylem vessels and fibers (**Figure 5C**). This phenomenon was already apparent at 1W and 2W after planting (**Figure 5C**). Paclobutrazol application, as opposed to GA_3_, did not induce fiber formation (**Figure 5C**). Cross-sections of SRs formed at 5W of development did not exhibit major differences between treatments except for formation of an irregular vascular cambium ring in GA_3_-treated plants (**Supplementary Figure S5**).

**Figure 6:**
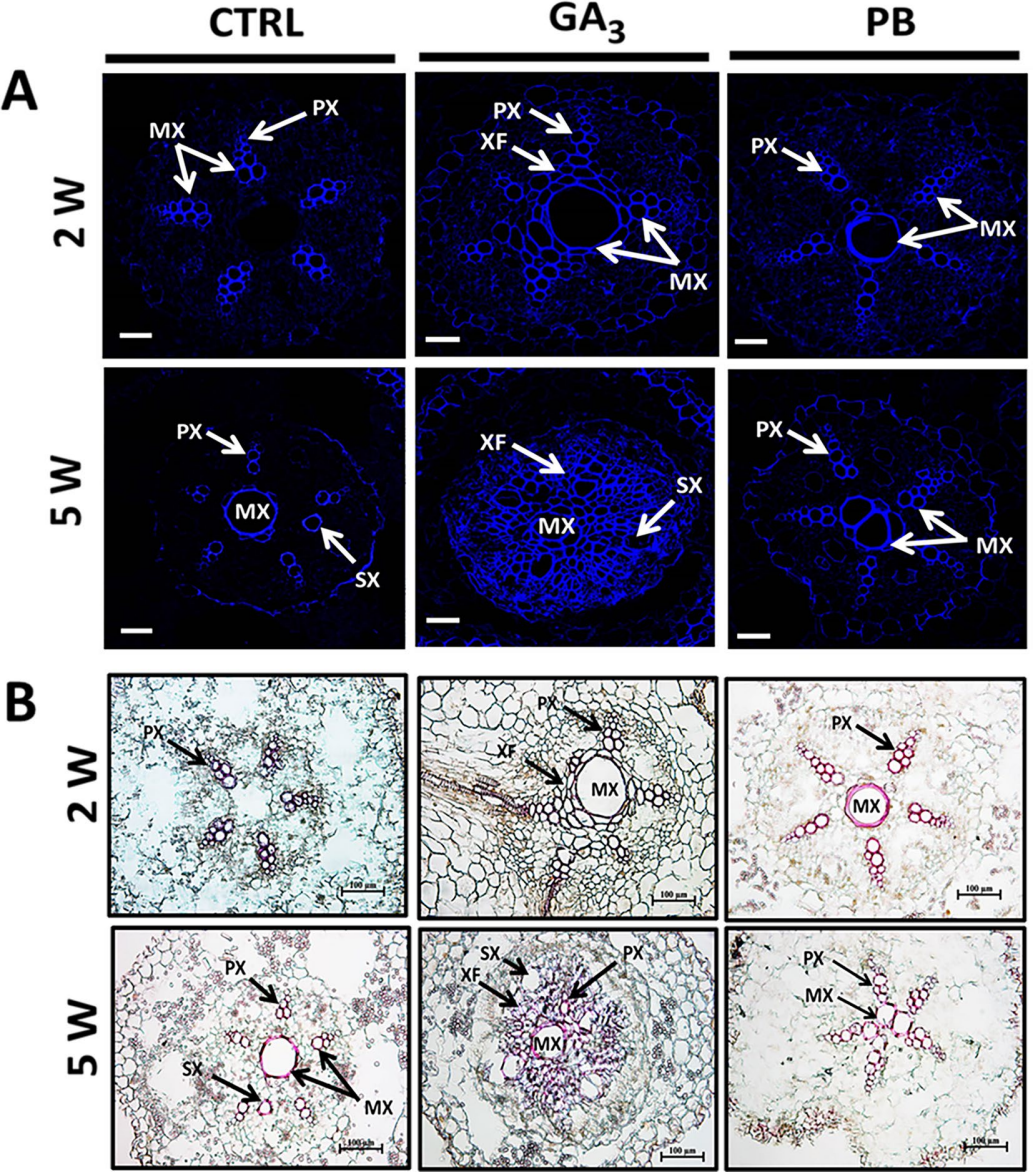
Effect of GA_3_ application on sweetpotato “*Georgia Jet*” root lignin accumulation as viewed by auto-fluorescence imaging **(A)** and phloroglucinol-HCl staining **(B)**. Representative cross-sections of adventitious roots sampled at two and five weeks (2W and 5W, respectively) after planting. Use of autofluorescence imaging (a nonspecific tool indicative of lignin and various phenolics) specifying secondary-wall material deposition in xylem showing blue fluorescence under UV excitation at 365 nm, points to lignin accumulation in xylem elements including proto-, meta-, and secondary xylem, and xylem fibers **(A)**. Phloroglucinol-HCl staining (being more specific and indicative of 4-O-linked hydroxycinnamyl aldehyde structures in lignins) stained lignified cells wall, where lignin deposition appears as pink-red color in proto-, meta-, and secondary xylem, and xylem fibers **(B)**. Presented sections represent six to eight roots sampled from individual plants. CTRL, Control (treated with water); GA_3_, application of 50 ppm gibberellic acid 3 for two weeks; PB, application of 5 ppm paclobutrazol for two weeks. PX, protoxylem; MX, metaxylem; SX, secondary xylem; XF, xylem fibers. Scale bar = 50 µm **(A)** and 100 µm **(B)**.

In order to follow the effect of gibberellin on lignification, we have used auto-fluorescence imaging (being a nonspecific tool indicative of lignin and various phenolics (Donaldson and Knox, 2012) as well as phloroglucinol staining (indicative of the 4-O-linked hydroxycinnamyl aldehyde structures in lignins) (Pomar et al., 2002) and results are presented in **Figure 6**. The results indicate higher lignification of root cross-sections induced by GA_3_ as well as PB treatments, as visualized by both auto-fluorescence and phloroglucinol staining (**Figures 6A, B**, respectively). Highest lignification/lignified area was evident in 2W and 5W samples following GA_3_ treatment. It should be noted that GA_3_ and PB treatments were stopped at 2W after planting, while their effect was obvious during the treatment period and several weeks afterwards. At 5W, the lignified area in GA_3_-treated roots was represented mainly by fibers, while in PB-treated roots it was represented by proto- and meta-xylem cells.

### Exogenous Application of GA_3_ Affects Starch Accumulation in Sweetpotato Roots

**Figure 7:**
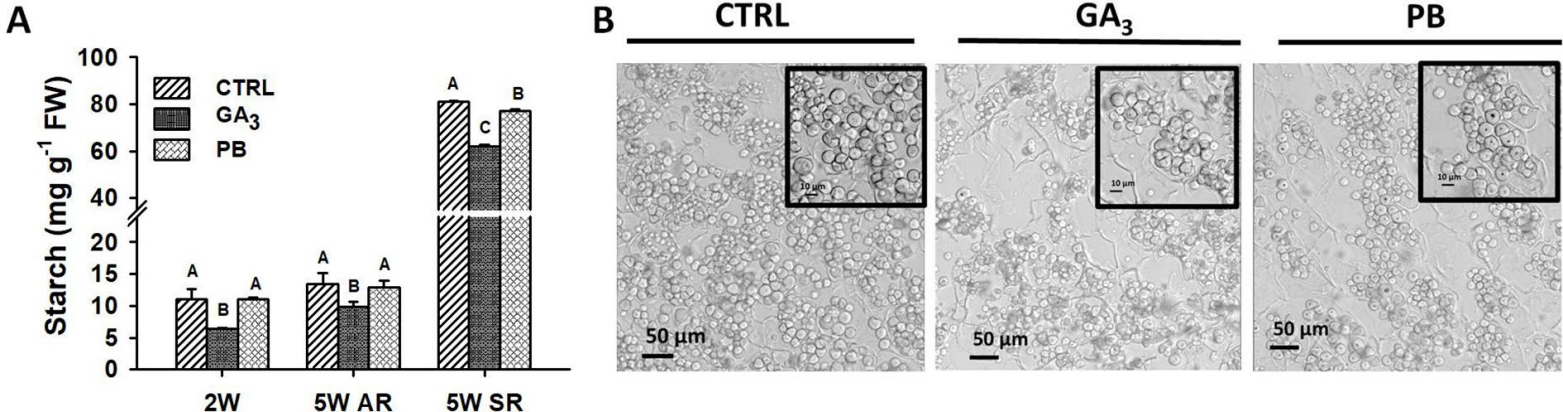
Effect of GA_3_ application on starch level **(A)**, and starch granules accumulation **(B)** in sweetpotato “*Georgia Jet*” roots. Starch level was quantified in roots collected at two and five weeks (2W and 5W, respectively) after planting. At 5W, both adventitious roots (AR) that did not develop into storage-root (SR), and SR were analyzed. Bars in **(A)** represent mean of three independent biological replicates (each representing roots pooled from four plants) ± SE. Significance analysis was performed by using student’s t-test (*P ≤ 0.05*), where unlike letters represent significant differences between treatments within a group. Cross sections of SR collected at 5W of development, representing six independent biological replicates (plants), are given in **(B)** as viewed under a confocal microscope focusing on starch granules. Scale bar = 50 µm and 10 µm (in inset). Control (treated with water); GA_3_, application of 50 ppm gibberellic acid 3 for two weeks; PB, application of 5 ppm paclobutrazol for two weeks.

Since starch accumulation is an important indication for formation of a SR, we investigated starch content at 2W (in ARs) and 5W (in ARs and SRs) after planting in nontreated and treated (GA_3_- and PB-treated) plant roots (**Figure 7A**). GA_3_ was found to significantly reduce starch content in both 2W and 5W AR samples. Interestingly, GA_3_ treatment showed a significant reducing effect on starch accumulation also in roots that developed into SRs. Paclobutrazol application did not show an effect on starch accumulation in ARs at either 2W or 5W after planting, while causing reduction in starch levels accumulating in SRs. Furthermore, GA_3_ and PB treatments caused reduced starch granule density in SRs as seen in cross-sections (**Figure 7B**).

### Exogenous Application of GA_3_ Influences the Expression Pattern of Genes Related to Gibberellin Biosynthesis and Regulation

**Figure 8:**
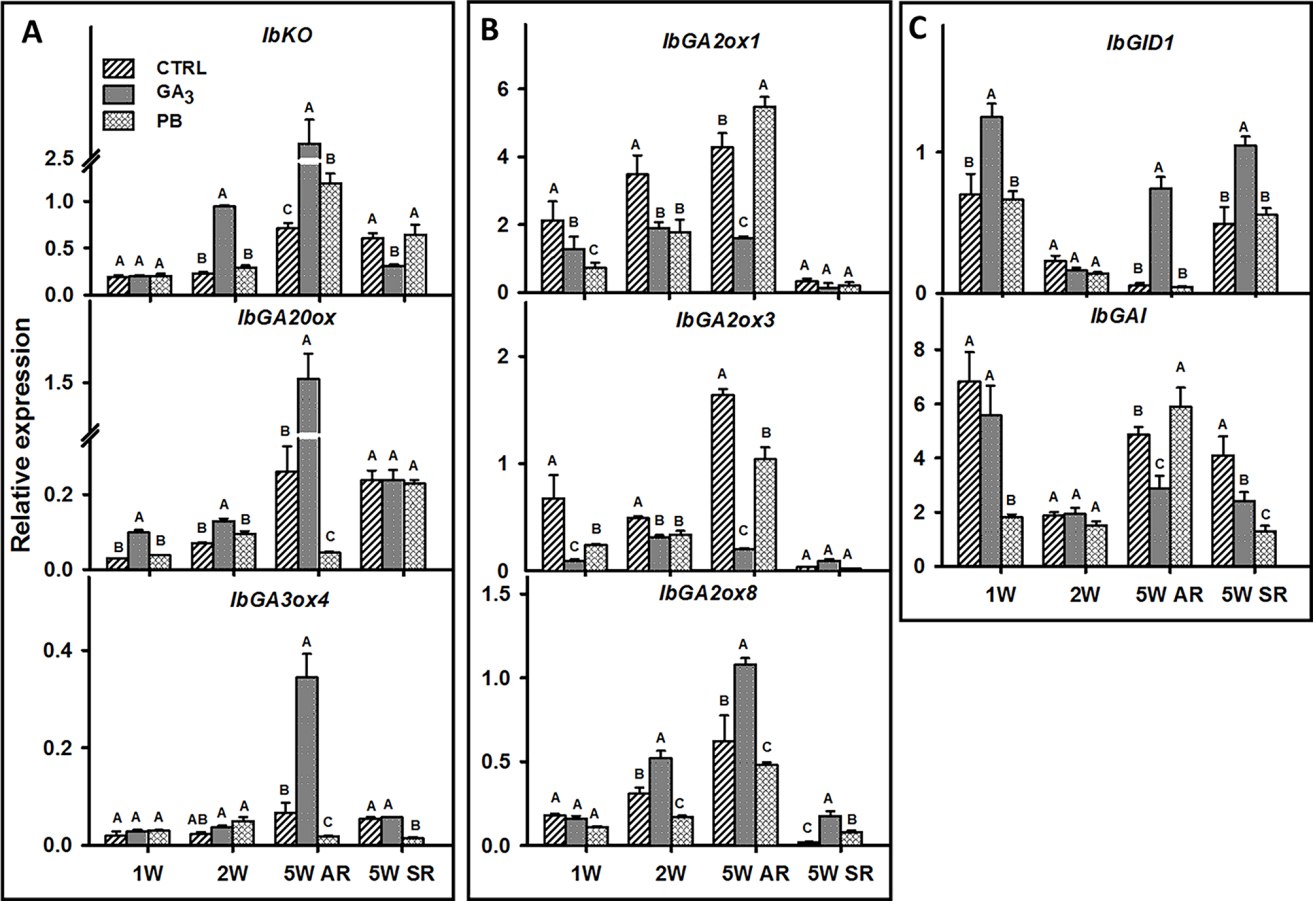
The effect of GA_3_ application on expression profiles of sweetpotato “*Georgia Jet*” orthologs/genes involved in gibberellin biosynthesis **(A)**, catabolism **(B)**, and signaling/regulation **(C)** in adventitious roots (AR) and storage-roots (SR). Expression/transcript levels were measured at 1W, 2W, and 5W after planting for AR and at 5W for SR, using qRT-PCR analyses, and values were normalized relative to the expression levels of phospholipase D1a. qRT-PCR data are mean (± SE) of three independent biological replicates (each representing roots pooled from four plants). Significance analysis was performed by using student’s t-test (*P ≤ 0.05*), where unlike letters represent significant differences between treatments within a group. Control (treated with water); GA_3_, application of 50 ppm gibberellic acid 3 for two weeks; PB, application of 5 ppm paclobutrazol for two weeks.

We identified sweetpotato orthologues for *Arabidopsis* genes involved in GA regulation, vascular development, lignin biosynthesis, meristem regulation, and starch biosynthesis, using the respective gene sequences/contigs derived by us previously by sequencing the *“Georgia Jet”* root transcriptome (Firon et al., 2013). The list of all sweetpotato contigs together with the respective *Arabidopsis* genes is presented in **Table 1**. The expression pattern of the following GA-related genes was followed during root development at 1W, 2W, and 5W: GA biosynthesis genes, ent-kaurene oxidase (*IbKO*), GA20-oxidase (*IbGA20ox*), and GA3-oxidase4 (*IbGA3ox4*) (**Figure 8A**); GA catabolizing genes, GA2-oxidase (*IbGA2ox1, IbGA2ox3*, and *IbGA2ox8*) (**Figure 8B**), and genes involved in GA signaling and regulation, GIBBERELLIN INSENSITIVE DWARF1 (*IbGID1*) and GIBBERELLIC ACID INSENSITIVE (*IbGAI*) (**Figure 8C**). The results show a significant increase in *IbKO* expression following GA_3_ application, exhibiting 4.1- and 3.6-fold increased transcript level at 2W and 5W ARs, respectively, compared with control (**Figure 8A**). Similarly, *IbGA20ox* expression increased significantly by GA_3_ in ARs harvested at 1W, 2W, and 5W, while *IbGA3ox4* expression increased mainly in 5W ARs (**Figure 8A**). The expression of the GA receptor gene, *IbGID*1, was also strongly upregulated by GA_3_ application, 1.8- and 13.4-fold at 1W and 5W ARs, respectively, while genes involved in GA catabolism (*IbGA2ox1* and *IbGA2ox3*) were down-regulated (**Figure 8B**). The expression of yet another GA2-oxidase (*IbGA2ox8*) was however upregulated by GA_3_ (1.7-fold in 2W and 5W ARs). Application of PB caused an opposite effect compared to GA_3_, downregulating expression of two GA biosynthesis genes (*IbGA20ox* and *IbGA3ox4*) as well as downregulating *IbGA2ox8* (evident in 2W and 5W ARs), while causing upregulation of *IbGA2ox1* (evident in 5W ARs). Expression of *IbGID1* was maintained similar to control (**Figure 8C**). The negative regulator of GA, *IbGAI*, showed variable responses to GA_3_ and PB, being opposite in 5W ARs (reduced expression by GA_3_ and upregulation by PB). Comparing between 5W ARs and SRs, similar expression patterns were observed for *IbGA2ox8* and *IbGID1* following GA_3_ and PB treatments, while expression of *IbGA20ox, IbGA2ox1*, and *IbGA2ox3* was found to stay similar in SRs across treatments.

**Table 1:** Sweetpotato (*Ipomoea batata) Georgia Jet* orthologues/genes investigated in our study.

*Ipomoea batata* contigs	*Ipomoea batata* gene	Source/NCBI accessions	*Arabidopsis* homolog	*Arabidopsis* gene name	Gene function	Gene Family
Gibberellin biosynthesis and signaling/regulation						
S_PBL_c17751	Kurene oxidase (*IbKO*)	Firon et al., 2013	AT5G25900	Ent-Kaurene oxidase 1 (*KO*)	Gibberellin biosynthesis	Various
S_PBL_c36353	Gibberellin 20-dioxygenase (*IbGA2Oox*)	Firon et al., 2013	AT4G25420	Gibberellin 20 oxidase 1 (*GA2Oox1*)	Gibberellin biosynthesis	
S_PBL_c51845	Gibberellin 3-beta-dioxygenase 4 (*IbGA3ox4*)	Firon et al., 2013	AT1G80330	Gibberellin 3 oxidase 4 (*GA3ox4*)	Gibberellin biosynthesis	
S_PBL_c23111	Gibberellin 2-oxidase 1 (*IbGA2ox1*)	Firon et al., 2013	AT1G78440	Gibberellin 2-oxidase 1 (*GA2ox1*)	Catalyzing bioactive GAs to inactive forms	
S_PBL_c32	Gibberellin 2-oxidase 3 (*IbGA2ox3*)	Firon et al., 2013	AT1G78440	Gibberellin 2-oxidase 1 (*GA2ox1*)	Catalyzing bioactive GAs to inactive forms	
S_PBL_c13427	Gibberellin 2-oxidase8 (*IbGA2ox8*)	Firon et al., 2013	AT5G58660	2-oxoglutarate (2OG) Fe(II)-dependent oxygenase	Catalyzing bioactive GAs to inactive forms	
S_PBL_c19593	Gibberellin insensitive dwarf1 (*IbGID1*)	Firon et al., 2013	AT3G63010	GA insensitive dwarf1b (*GID1B*)	Gibberellin receptor	
S_PBL_c1873	GA-insensitive (*IbGAI/RGA2*)/DELLA protein	Firon et al., 2013	AT1G14920	Gibberellic acid insensitive (*GAI*)	Repressor of gibberellin responses	
Regulators of vascular development						
S_PBL_c36855	VASCULAR RELATED NAC-DOMAIN PROTEIN 075 (*IbNAC075*)	Firon et al., 2013	AT4G29230	NAC DOMAIN CONTAINING PROTEIN 75, (*NAC075*)	Secondary wall biosynthesis in xylem vessels.	NAC-DOMAIN PROTEIN
S_PBL_c32341	VASCULAR RELATED NAC-DOMAIN PROTEIN 7 (*IbVND7*)	Firon et al., 2013	AT1G71930	VASCULAR RELATED NAC-DOMAIN PROTEIN 7 (*VND7*)	Xylem vessel differentiation	
S_PBL_c24252	SECONDARY WALL-ASSOCIATED NAC DOMAIN 2 (*IbSND2*)	Firon et al., 2013	AT4G28500	SECONDARY WALL-ASSOCIATED NAC DOMAIN PROTEIN 2, (*SND2*)	Secondary wall biosynthesis in xylem fibers	
S_PBL_c504	XYLEM NAC DOMAIN1 (*IbXND1*)	Firon et al., 2013	AT5G64530	XYLEM NAC DOMAIN 1 (*XND1*)	Negative regulator of xylem vessel differentiation and secondary wall thickening	
S_PBL_c4628	VND-INTERACTING2 (*IbVNI2*)	Firon et al., 2013	AT5G13180	NAC DOMAIN CONTAINING PROTEIN 83 (*NAC083*), VND-INTERACTING 2 (*VNI2*)	Negative regulator of xylem vessel differentiation and secondary wall thickening	
S_PBL_c17476	VND-INTERACTING2 like (*IbVNI2-like*)	Firon et al., 2013	AT1G62700	NAC DOMAIN CONTAINING PROTEIN 83-like (*NAC083*-like), VND-INTERACTING 2 (*VNI2*-like)	Negative regulator of xylem vessel differentiation and secondary wall thickening	
Lignin biosynthesis						
S_PBL_c2312	Phenylalanine ammonia lyase (*IbPAL*)	Firon et al., 2013	AT2G37040	Phenylalanine ammonia lyase 1 (*PAL1*)	Lignin biosynthesis	Various
S_PBL_c7605	Cinnamate 4-hydroxylase (*IbC4H*)	Firon et al., 2013; GQ373157	AT2G30490	Cinnamate-4-hydroxylase (*C4H*)	Lignin biosynthesis	
S_PBL_c18044	4-Coumarate-CoA ligase (*Ib4CL*)	Firon et al., 2013	AT1G51680	4-Coumarate : Coa ligase 1 (*4CL*)	Lignin biosynthesis	
S_PBL_c17752	Hydroxycinnamoyl transferase (*IbHCT*)	Firon et al., 2013; AB576768	AT5G48930	Hydroxycinnamoyl transferase (*HCT*)	Lignin biosynthesis	
S_PBL_c2944	Caffeoyl-CoA-O-methyltransferase (*IbCCoAOMT*)	Firon et al., 2013; EU250002	AT4G34050	Caffeoyl coenzyme a o-methyltransferase 1 (*CCoAOMT*)	Lignin biosynthesis	
S_PBL_lrc53688	Cinnamyl alcohol dehydrogenase (*IbCAD*)	Firon et al., 2013; GU380306	AT4G39330	Cinnamyl Alcohol Dehydrogenase (*CAD*)	Lignin biosynthesis	
Class I knotted 1-like						
S_PBL_c8137	Class I knotted1-like homeobox (KNOX1) (*IbKN2*)	Firon et al., 2013; AB283028	AT4G08150	BREVIPEDICELLUS (*KNAT1*)	Meristematic maintenance	KNOX1
S_PBL_c31412	Class I knotted1-like homeobox (KNOX1) (*IbKN3*)	Firon et al., 2013; AB283029	AT4G08150	BREVIPEDICELLUS (*KNAT1*)	Meristematic maintenance	
Carbohydrate metabolism and starch biosynthesis						
S_PBL_c543	Sucrose synthase (*IbSuSy*)	Firon et al., 2013; EU908020	AT3G43190	Sucrose synthase 4 (*SUS4*)	Sucrose metabolism	Various
S_PBL_c20112	Phosphoglucomutase (*IbPGM*)	Firon et al., 2013	AT1G70730	Phosphoglucomutase 2 (*PGM2*)	Interconversion of glucose 1-phosphate and glucose 6-phosphate	
S_PBL_c18129	ADP-glucose pyrophosphorylase alpha subunit (*IbAGPa1*)	Firon et al., 2013; KJ365312, JQ797696, Z79635, X83498, Z46756, AY544766	AT5G48300	ADP-glucose pyrophosphorylase small subunit	Starch biosynthesis	
S_PBL_c54187	ADP-glucose pyrophosphorylase beta subunit (*IbAGPb1A*)	Firon et al., 2013; JQ797698, JQ797692, AB271013, AF068260, AJ249257, AJ249256, AJ252316, AJ245392, AB071976	AT1G27680	ADP-glucose pyrophosphorylase large subnuit	Starch biosynthesis	
S_PBL_c3042	Granule-bound starch synthase (*IbGBSS*)	Firon et al., 2013; AB524722/23/24/25/26/27/28, AB071604	AT1G32900	Granule bound starch synthase 1 (*GBSS1*)	Starch biosynthesis	
S_PBL_c1370	Starch phosphorylase (*IbSP*)	Firon et al., 2013; L25626, M64362	AT3G29320	ALPHA-glucan phosphorylase 1 (*PHS1*)	Phosphorolytic degradation of starch	

### Expression of Genes Involved in Regulation of Vascular Development and Lignin Biosynthesis in Response to GA_3_ Application

**Figure 9:**
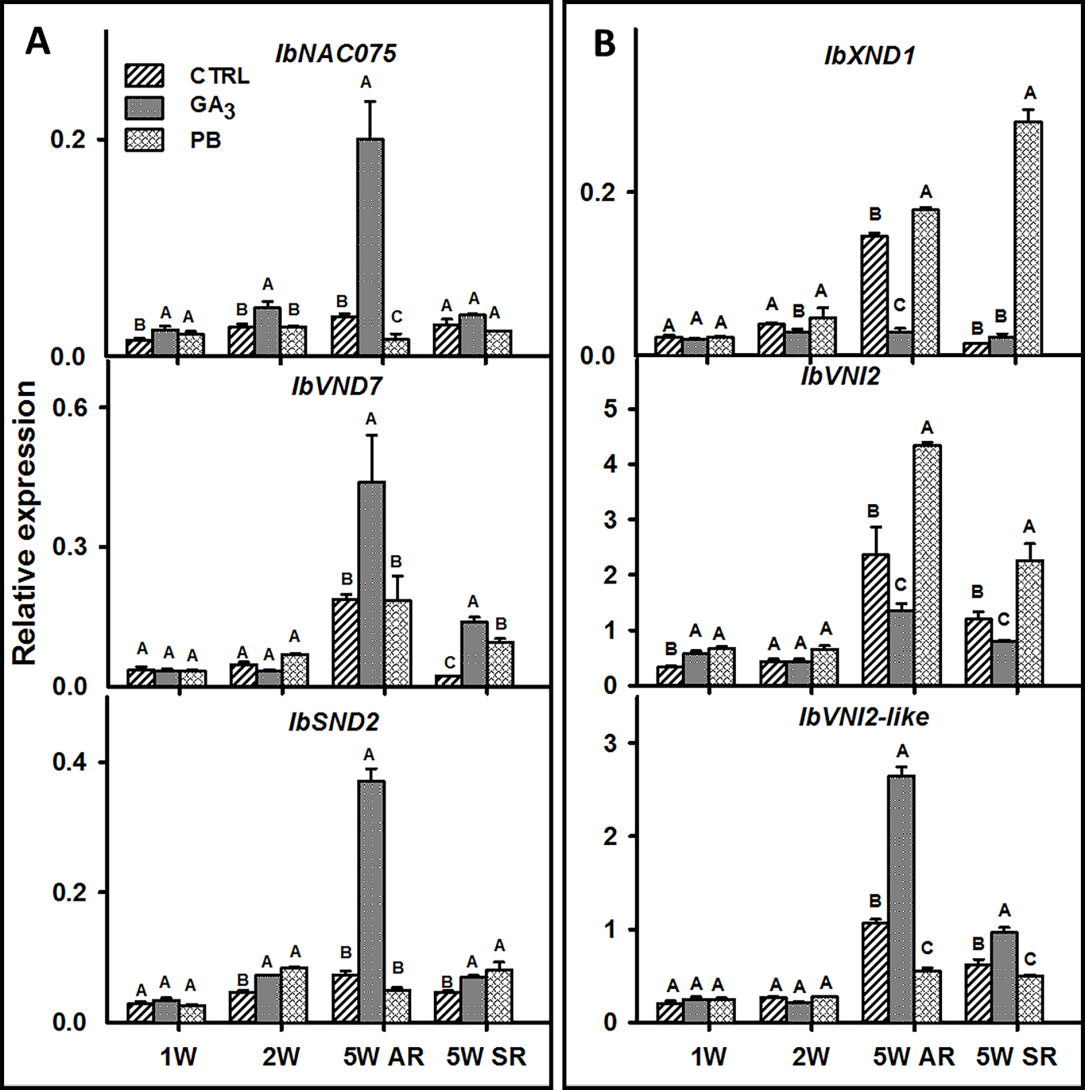
The effect of GA_3_ application on expression profiles of sweetpotato “*Georgia Jet*” potential positive **(A)** and negative **(B)** regulators of vascular development in adventitious roots (AR) and storage-roots (SR). Expression/transcript levels were measured at 1W, 2W, and 5W after planting for AR and at 5W for SR, using qRT-PCR analyses, and values were normalized relative to the expression levels of phospholipase D1a. qRT-PCR data are mean (± SE) of three independent biological replicates (each representing roots pooled from four plants). Significance analysis was performed by using student’s t-test (*P ≤ 0.05*), where unlike letters represent significant differences between treatments within a group. Control (treated with water); GA_3_, application of 50 ppm gibberellic acid 3 for two weeks; PB, application of 5 ppm paclobutrazol for two weeks.

To look for genes involved in the regulation of root xylem development and vascular lignification following GA_3_ application, we identified the following sweetpotato orthologues of the respective *Arabidopsis* genes: *IbNAC075* (upstream regulator of VND7) (Endo et al., 2015), *IbVND7* (involved in xylem vessel differentiation) (Yamaguchi et al., 2008), *IbSND2* (regulator of genes involved in secondary wall development) (Hussey et al., 2011), *IbXND1* (negative regulator of xylem formation) (Tang et al., 2018), *IbVNI2* (transcriptional repressor of VND7) (Yamaguchi et al., 2010), and *IbVNI2*-like (**Table 1**) and their transcript profiles were investigated (**Figure 9**). The expression of all tested positive regulators (**Figure 9A**) was induced by GA_3_ treatment as evident in ARs sampled at 5W after planting, while expression of *IbNAC075* was upregulated earlier, already at 2W (**Figure 9A**). The expression of the negative regulators, *IbXND1* and *IbVNI2*, was, however, downregulated by GA_3_ (5.2- and 1.7-fold, respectively, in 5W ARs) (**Figure 9B**). Expression of *IbVNI2-like* was upregulated (2.5-fold) by GA_3_ application when tested at 5W after planting. Application of PB exhibited an opposite effect as compared to GA_3_ on expression of most tested genes as evident in the 5W samples (showing down- and up-regulated expression of *IbNAC075* and *IbXND1*, respectively; **Figure 9**).

**Figure 10:**
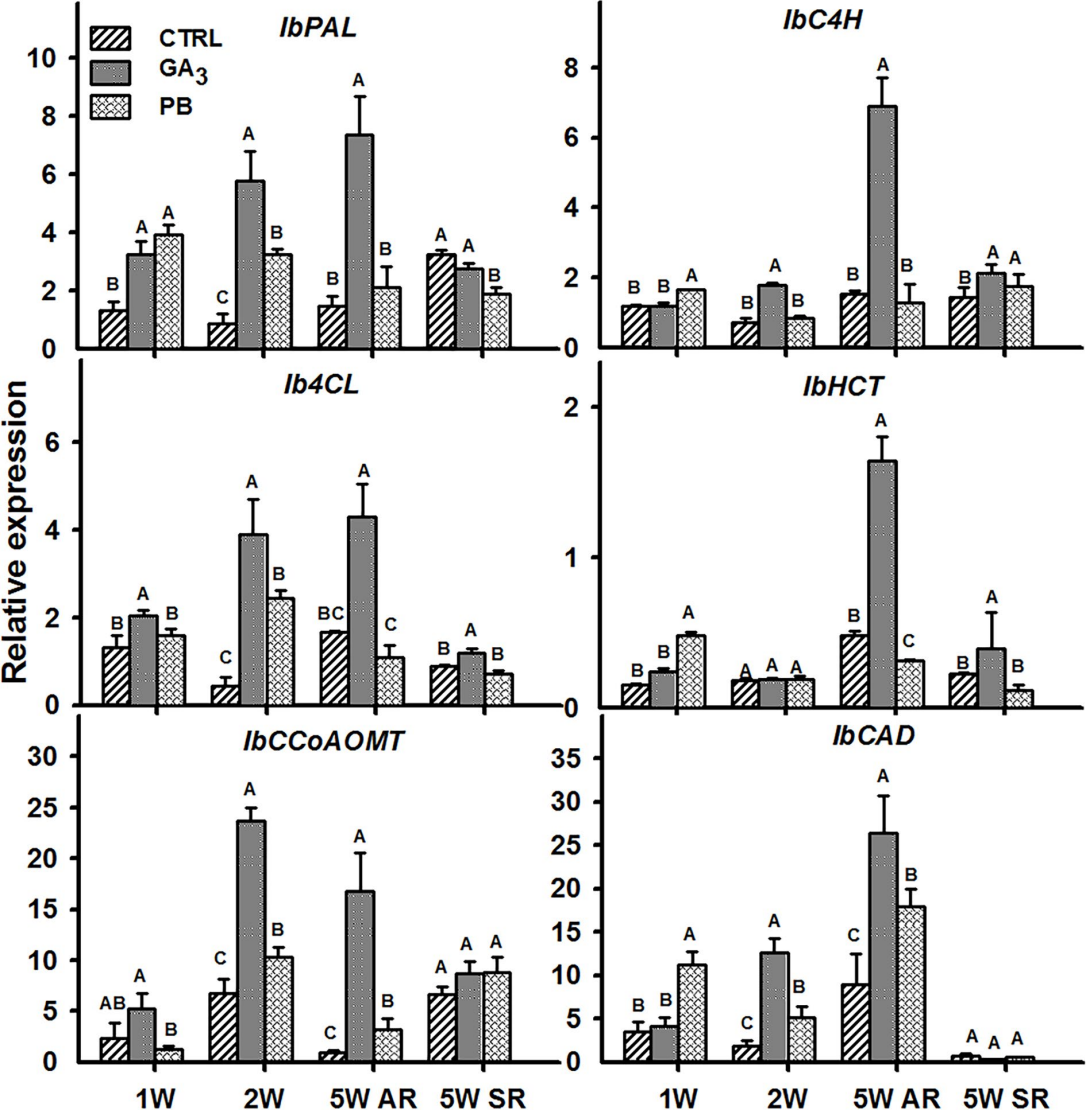
The effect of GA_3_ application on expression profiles of sweetpotato “*Georgia Jet*” orthologues/genes of lignin biosynthesis in adventitious roots (AR) and storage-roots (SR). Expression/transcript levels were measured at 1W, 2W, and 5W after planting for AR and at 5W for SR, using qRT-PCR analyses, and values were normalized relative to the expression levels of phospholipase D1a. qRT-PCR data are mean (± SE) of three independent biological replicates (each representing roots pooled from four plants). Significance analysis was performed by using student’s t-test (*P ≤ 0.05*), where unlike letters represent significant differences between treatments within a group. Control (treated with water); GA_3_, application of 50 ppm gibberellic acid 3 for two weeks; PB, application of 5 ppm paclobutrazol for two weeks.

Lignin biosynthesis starts with deamination of phenylalanine by PAL, followed by a series of reactions involving C4H, 4CL, HCT, CCoAOMT, and CAD (Kao et al., 2002; Raes et al., 2003). All tested sweetpotato lignin biosynthesis genes, exhibited significantly elevated transcript levels following application of GA_3_ (**Figure 10**). This upregulation was evident in ARs sampled at 2W and 5W after planting and, in the case of *IbPAL* and *Ib4CL*, already at 1W after planting. This gene expression pattern parallels the elevated lignification observed in tissue sections (**Figure 6**). The expression of all lignin-biosynthesis genes (except *IbHCT*), following PB treatment was, however, significantly lower as compared to roots of GA_3_-treated plants (as seen in the 2W and 5W AR samples; **Figure 10**).

**Figure 11:**
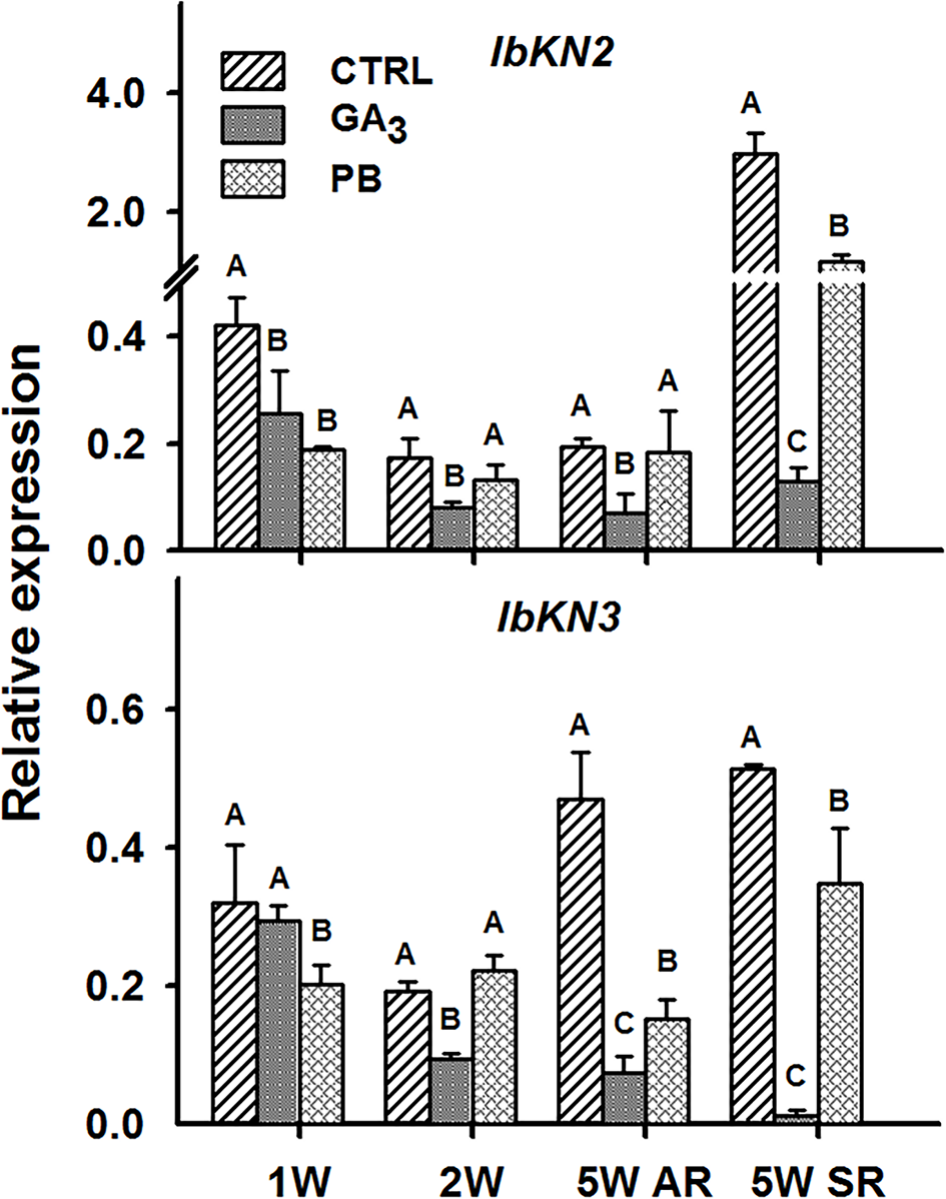
The effect of GA_3_ application on expression profiles of sweetpotato “*Georgia Jet*” class I knotted 1-like orthologs/genes in adventitious roots (AR) and storage-roots (SR). Expression/transcript levels were measured at 1W, 2W, and 5W after planting for AR and at 5W for SR, using qRT-PCR analyses, and values were normalized relative to the expression levels of phospholipase D1a. qRT-PCR data are mean (± SE) of three independent biological replicates (each representing roots pooled from four plants). Significance analysis was performed by using student’s t-test (*P ≤ 0.05*), where unlike letters represent significant differences between treatments within a group. Control (treated with water); GA_3_, application of 50 ppm gibberellic acid 3 for two weeks; PB, application of 5 ppm paclobutrazol for two weeks.

In the context of lignification, it was of high interest to test the transcript profiles of two class I knotted 1-like transcription factors, orthologues of the *Arabidopsis BP* gene, *IbKN2* and *IbKN3*, following GA_3_ treatment. The *BP* gene was previously shown to regulate lignin accumulation and lignin biosynthesis genes in *Arabidopsis* (Mele et al., 2003). Expression of both *IbKN2* and *IbKN3* followed a similar pattern, exhibiting significantly down-regulated transcript levels following GA_3_ treatment (**Figure 11**). The inhibitory effect of GA_3_ on *IbKN2*/*3* expression was evident also in the sampled SRs. Paclobutrazol did not show a pronounced effect on the expression of these genes at 2W, although at 5W, when tested in ARs, it caused reduced expression of *IbKN3* and exhibited no effect on *IbKN2* (**Figure 11**). It is worth mentioning that *BP* orthologues were previously indicated as markers of cambial cells and were shown to regulate development and maintenance of the shoot apical meristem in various plants (Vollbrecht et al., 2000; Schrader et al., 2004).

### Expression of Genes Involved in Starch Biosynthesis in Response to GA_3_ Application

**Figure 12:**
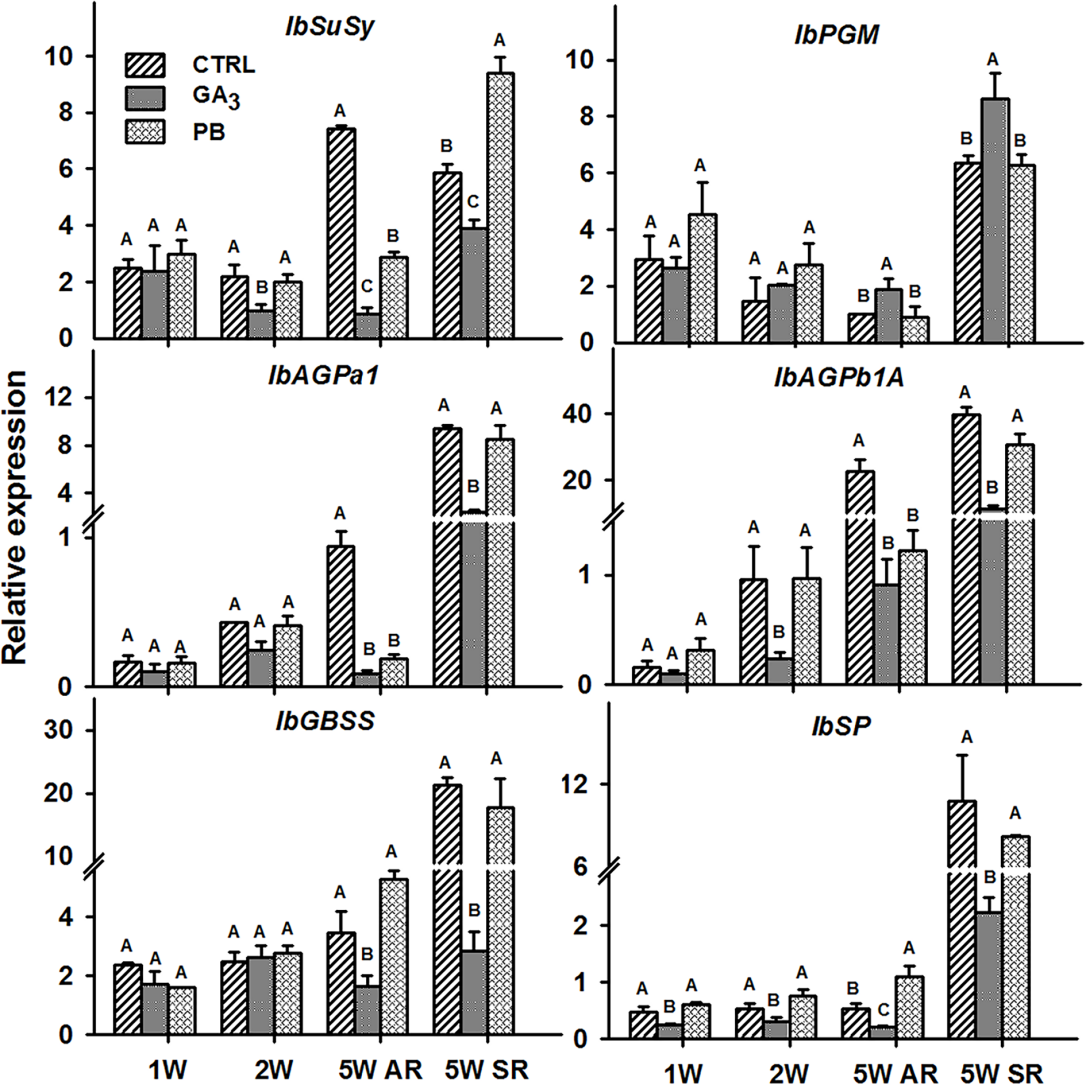
The effect of GA_3_ application on expression profiles of sweetpotato “*Georgia Jet*” carbohydrate metabolism and starch biosynthesis orthologs/genes in adventitious roots (AR) and storage-roots (SR). Expression/transcript levels were measured at 1W, 2W and 5W after planting for AR and at 5W for SR, using qRT-PCR analyses and values were normalized relative to the expression levels of phospholipase D1a. qRT-PCR data are mean (± SE) of three independent biological replicates (each representing roots pooled from four plants). Significance analysis was performed by using student’s t-test (*P ≤ 0.05*), where unlike letters represent significant differences between treatments within a group. Control (treated with water); GA_3_, application of 50 ppm gibberellic acid 3 for two weeks; PB, application of 5 ppm paclobutrazol for two weeks.

The following six sweetpotato genes, known to be involved in carbohydrate metabolism and starch biosynthesis were studied for their expression behavior following GA_3_ and PB treatments: Sucrose synthase (*IbSuSy*), phosphoglucomutase (*IbPGM*), ADP-glucose pyrophosphorylase small and large subunits (*IbAGPa1* and *IbAGPb1A,* respectively), granule-bound starch synthase (*IbGBSS*), and starch phosphorylase (*IbSP*) (**Figure 12**). Expression of all tested genes, except *IbPGM*, was reduced by GA_3_ application. Expression of *IbSuSy* and *AGPb1A* was down-regulated 2.2- and 4.0-fold, respectively, as early as 2W, and 8.7- and 24.7-fold, respectively, in 5W ARs. In addition, GA_3_ application caused down-regulation in expression of *IbGBSS* and *IbSP* (2.1- and 2.6-fold, respectively, in 5W ARs). The inhibitory effect of GA_3_ on expression levels of all tested genes (except *IbPGM*) was evident also in SRs. Paclobutrazol treatment did not cause a significant change in expression in either of the tested carbohydrate metabolism genes at 2W of development, while expression of *IbSuSy, IbAGPa1* and *IbAGPb1A* was reduced when tested in 5W ARs (**Figure 12**).

## Discussion

### Root System Architecture During the First 2 Weeks of Root Development Is Linked to Transition of the Sweetpotato Adventitious Root Into a Storage-Root

The aim of the current study was to gain an understanding of the key physiological and molecular events/mechanisms that mark the developmental transition of the sweetpotato AR into a storage organ. The work was based upon previous findings from our laboratory (in collaboration with the laboratories of A. Villordon and D. LaBonte) demonstrating that (i) the initial ARs that develop from stem-cutting possess the necessary requirements for forming SRs (Villordon et al., 2009); (ii) SR initiation phase spans the first two weeks after planting (Villordon et al., 2009). We made use of the plant hormone GA, shown previously to affect RSA (Gou et al., 2010) and lignification (Wang et al., 2017; Duan et al., 2019), in order to study its effect on sweetpotato root development, SR formation, lignin accumulation, and the expression levels of lignin-biosynthesis, and carbohydrate metabolism/starch-biosynthesis genes as well as potential upstream regulators. This enabled us to get an insight into what processes and genes are involved in SR initiation.

Exogenous application of GA_3_ to sweetpotato plants during the first two weeks of development caused elevated levels of bioactive and non-bioactive GAs in the roots together with an increase in the plant’s stem length. Such an effect was previously reported in other systems like dandelion (Kim et al., 2009) and apple (Zhang et al., 2016). Increased levels of different GAs in response to GA_3_ application point to either conversion of GA_3_ to the other bioactive forms (by a yet unknown mechanism), downregulation of catabolism and/or possible upregulation of the GA biosynthesis pathway by a feedforward mechanism. Transcript levels of sweetpotato orthologues of the *Arabidopsis* GA biosynthesis genes *IbKO*, *IbGA20ox*, and *IbGA3ox* and the receptor *IbGID1* were highly upregulated by GA_3_, while transcript levels of genes encoding enzymes of GA deactivation (*IbGA2ox1* and *IbGA2ox3*) were found to be downregulated, matching the induced endogenous GAs levels observed in our study and pointing to the potential existence of a feedforward mechanism. However, contrary to our findings, GA treatment was previously shown to downregulate the expression of *GA20ox* and upregulate expression of *GA2ox* (Thomas et al., 1999), and expression of GA biosynthesis genes was found to be elevated in a GA-deficient background and to be downregulated after application of bioactive GAs (Thomas et al., 1999; Yamaguchi, 2008 and references therein). Additional experiments are thus required in order to better understand the regulation of GA homeostasis in this unique system of sweetpotato AR development. Another interesting issue that requires additional study is the potential cross-talk and transport of GAs between shoot and root. Paclobutrazol, that functions as an inhibitor of GA biosynthesis by inhibiting oxidation of ent-kaurene into ent-kaurenoic acid (Rademacher, 2000), caused, as expected, reduction in all tested root GAs levels accompanied by reduction in sweetpotato stem length.

Exogenous application of GA_3_ caused a significant reduction in most tested RSA parameters, measured during the first two weeks after planting (SR initiation phase). Inhibition of root growth by GA_3_ treatment was recorded in other systems like carrot (Wang et al., 2015b; Wang et al., 2017). These inhibitory effects of GA on sweetpotato LR development and RSA were linked with a significant reduction in SR formation (SR number) and SR characteristics (diameter and weight). Thus, GA_3_ application affected both, the capacity of ARs to become SRs, and the further development and “bulking” of the small number of SRs that were formed. These results point to root architecture attributes during the first two weeks of plant development as being important and as playing a role in the developmental transition of the AR into a SR. Indeed, Villordon and Clark (2014) and Villordon et al. (2014) stressed the importance of root architecture in root (sweetpotato, cassava) and tuber (potato) crop productivity. It was previously demonstrated that ARs that showed evidence for SR initiation had higher values for LR attributes such as LR number, length, and surface area (Villordon et al., 2012).

The effect of PB on most tested root system parameters and on SR production was similar to that of GA_3_ in spite of its antagonistic effect on endogenous root GA levels. These results may point to (i) involvement of other factors besides GA in the effect of PB application or (ii) need for a delicate control of GA levels (not too much and not too little) in sweetpotato roots for enabling root system development. It should be mentioned in this context that other hormones, like auxin and cytokinin, are known to be involved in root growth and development and cross-talk between GA and different hormones was documented in various systems (Ogawa et al., 2003; Pacifci et al., 2015). In carrot root, for example, it was demonstrated that transcript levels of genes related to auxin, cytokinin, abscisic acid (ABA), and brassinolides were altered in response to increased or reduced gibberellins (Wang et al., 2015b). In addition, an organ-specific regulation of hormone-related genes in response to GA or PB was suggested (Wang et al., 2015b). Nonetheless, these results further substantiate the link between LR development and the capacity of the AR for SR formation (both being inhibited by PB application).

### Gibberellin Has a Central Role in the Upregulation of Sweetpotato Root Xylem Formation and Lignification, and Downregulation of Starch Biosynthesis—The Suggested Events Leading to Inhibition of Storage-Root Formation

Gibberellin was previously demonstrated to promote xylogenesis, induction of fiber production and fiber length, as well as lignification, in numerous plant systems including tobacco, carrot, and hybrid aspen (Eriksson et al., 2000; Biemelt et al., 2004; Dayan et al., 2010; Wang et al., 2017). In the presented work, in parallel to its promoting effect on xylem development and fiber formation, GA_3_ application caused induced lignification. Similar findings were reported recently in carrot taproot, demonstrating increased lignification following GA_3_ treatment (Wang et al., 2017). Exogenous application of PB, as opposed to GA_3_, did not induce secondary xylem and fiber formation, and its induction of lignification was less pronounced. Paclobutrazol, in spite of its inhibitory effect on accumulation of bioactive GAs in the sweetpotato root system, exhibited a similar effect to GA_3_ with respect to enhancing significantly the number of root xylem vessels. Additional studies are thus needed in order to better understand the effect of PB on xylem development and lignin accumulation, as compared to GA_3_.

Togari (1950) proposed a direct link between root lignification and SR initiation, suggesting that lignification inhibits SR formation. Our results, together with studies in additional systems of SR development, like carrot and cassava, further strengthen the idea that lignification prevents SR formation. Such a relationship between stele lignification and inability of ARs to develop into SRs was pointed out in sweetpotato in additional studies (Wilson and Lowe, 1973; Belehu et al., 2004). Furthermore, transcription profiling in sweetpotato initiating SRs indicated down-regulation of lignin biosynthesis genes and up-regulation of carbohydrate metabolism and starch biosynthesis genes (Firon et al., 2013).

In the current study, GA_3_ application was found to cause both, upregulation of lignin accumulation and downregulation of starch levels in sweetpotato roots, being linked to inhibition of SR formation. We have previously suggested that major events involved in SR initiation are the reduction of carbon flow towards phenylpropanoid synthesis and its delivery into carbohydrate metabolism and starch biosynthesis (Firon et al., 2013). In the present work, GA_3_ may cause the opposite effect, namely reduction of carbon flow toward carbohydrate metabolism and delivery into phenylpropanoid and lignin synthesis. Gibberellin may also cause starch degradation as reported in *Arabidopsis* seeds where GA induced expression of an α-amylase gene in developing seeds (Kim et al., 2005).

### Does the GA Inhibitory Effect on Storage-Root Development Operate *Via* Upregulation of Specific NAC-Domain Transcription Factors?

The results of the current study point to GA as having a central role in regulating the main processes taking place during AR root development into either a lignified root or a storage organ. However, the molecular mechanisms underlying the regulation of these processes are not yet clear. In *Arabidopsis*, the NAC-domain transcription factors, NAC075 and VND7, are master regulators of xylem development and secondary wall deposition including lignin (Endo et al., 2015). VND7, in turn, can regulate downstream NAC-domain genes like *SND2* (Hussey et al., 2011), being upregulated by NAC075 (Endo et al., 2015), and inhibited by VNI2 (Yamaguchi et al., 2010) These genes, together with *XND1* (negative regulator of xylem lignocellulose synthesis; Zhao et al., 2008) are thus good candidates to serve as regulators for mediating the GA effect on xylem development and lignin accumulation in sweetpotato roots. In accordance with increased root xylem development and lignification levels, GA_3_-treated plants exhibited induced transcript levels of the sweetpotato orthologues of *Arabidopsis NAC075* and *VND7* (*IbNAC075* and *IbVND7*) genes, a corresponding increase in expression levels of *IbSND2*, as well as downregulation of *IbVNI2* and *IbXND1*. Paclobutrazol application, opposed to GA_3_, caused significant down-regulation of *IbNAC075* transcript levels. These results point to *IbNAC075* and *IbVND7* as potential master switches of root xylem proliferation and lignin biosynthesis, marking them as important candidates for future studies. Interestingly, *MeVND7* was found in cassava to be expressed in fibrous/lignified roots and not in SRs and was suggested as a regulator of xylem development (Siebers et al., 2017).

Previous studies demonstrated a correlation between changes in transcript levels of lignin biosynthesis genes and lignin accumulation (Zhong and Ye, 2009). In the system of carrot SR formation, it was recently found that exogenous GA enhanced lignin accumulation and induced the transcript levels of lignin biosynthesis genes, including *PAL*, *4CL*, *CCoAOMT*, and *CAD* (Wang et al., 2017). Highly increased levels of all tested sweetpotato orthologues of *Arabidopsis* lignin biosynthesis genes was detected in the sweetpotato root system, following GA_3_ application, correlating with the observed induction in lignin accumulation. Such a correlation between lignin levels and gene expression of lignin biosynthesis genes (including *IbPAL*, *IbCL*, and *IbCAD*) was previously shown by us to exist in sweetpotato roots, when comparing between the transcriptome of lignified roots and roots exhibiting SR initiation (marked by development of “anomalous cambial cells”; Firon et al., 2013).

Another transcription factor that may serve as a good candidate for regulating sweetpotato root development and mediating the GA effect in yet another way is the *KNOX1* gene. Interestingly, cross-talk between GA and *KNOX* genes was previously demonstrated in various systems (Hay et al., 2002). Knotted 1-like transcription factors are known regulators involved in the establishment and maintenance of plant meristems, found to be highly expressed in poplar cambial samples (Schrader et al., 2004) and to exhibit upregulated transcript levels in initiating SRs compared to lignified non-SRs (Firon et al., 2013). Such an interaction between GA and KNOX may affect the balance between lignification, on one hand, and meristem development, on the other hand, and may be highly relevant to the issue of SR formation. Mele et al. (2003) suggested in *Arabidopsis* that a *KNOX1* gene, *BP*, regulates the lignin pathway, repressing premature cell differentiation. Hay et al. (2002) pointed out that reduced activity of the GA pathway promotes meristematic activity in *Arabidopsis* leaves. Such a transcription factor is thus a good candidate for serving as a regulator of SR development, operating at an intersection between downregulation of lignification and enabling upregulation of root cambium development. In accordance with this hypothesis, GA_3_ application caused a significant reduction in transcript levels of both *IbKN2* and *IbKN3* genes. Furthermore, GA_3_ treatment caused significant downregulation of carbohydrate metabolism and starch biosynthesis genes including genes encoding the small and large subunits (*IbAGPa1* and *IbAGPb1A*) of the regulatory step in starch biosynthesis, conversion of glucose 1-phosphate and ATP to ADP-glucose and pyrophosphate by AGPase (Ballicora et al., 2004). It should be noted that Liebsch et al. (2014) showed that *BP* and *STM* weak mutants inhibit fiber formation in *Arabidopsis* hypocotyl and suggested that in the cambium, compared to the shoot meristem, *KNOX* genes (*BP* and *STM*) may have opposite consequences caused through different mechanisms. The effect of different levels of KNOX activity should be further studied in a variety of systems in order to better understand their function.

**Figure 13:**
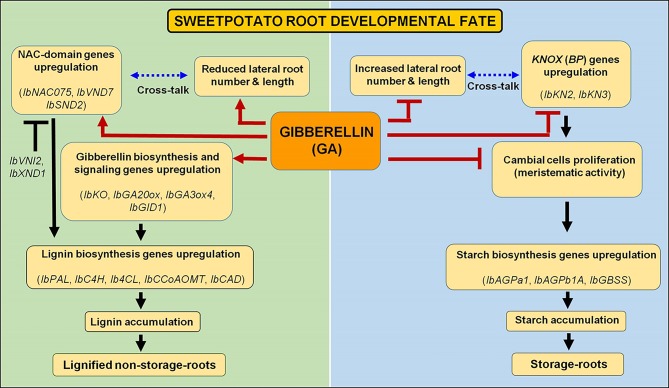
Schematic presentation showing a central role for gibberellin (GA) in formation of either lignified non-storage roots or storage roots. Gibberellin enhances xylem development and proliferation, and lignin accumulation, following the upregulation of NAC-domain genes (regulators of vascular development), and lignin biosynthesis genes, together with reducing root system architecture and lateral root development, bringing about the formation of lignified non-storage roots. At the same time, GA inhibits transcript levels of *KNOX* genes and of genes involved in starch biosynthesis, followed by inhibition of starch accumulation, causing inhibition of the developmental pathway that brings about storage-root formation. Genes considered significant to the respective processes are indicated.

The significance of the current findings is illustrated by highlighting potential regulators/regulatory mechanisms that control SR formation in sweetpotato to be used for enhancing yield under variable growth conditions. A model is suggested (**Figure 13**), serving as a basis for further studies. According to this model, GA has a central and active role in upregulating the upstream master switches of xylem development and lignin biosynthesis, *IbNAC075* and *IbVND7* transcription factors, which in turn cause upregulation of yet another NAC domain containing regulator, *IbSND2*. This cascade leads to upregulation of lignin biosynthesis genes, causing high lignification of the AR and development of a non-SR. In parallel, GA downregulates *KNOX1* genes and starch biosynthesis genes, highly reducing starch accumulation and inhibiting SR formation. GA also affects sweetpotato root system architecture, reducing LR number and LR length by an unknown mechanism. The results suggest that regulation of these processes in the root system is achieved at transcript level, and point to the respective genes as a valuable database for further studies, looking into/identifying the regulatory gene network. In addition, it is concluded that the response of sweetpotato roots to PB is complex, being opposite to GA_3_ in some parameters (fiber formation, gene expression of *IbGA20ox1*, *IbGA3ox4*, *IbGA2ox1*, *IbGAI*, *IbNAC075*, *IbXND1*, and *IbHCT*) and similar to that of GA_3_, but attenuated, in other factors (lignification, SR number). These results may reflect the complex interactions/influence of PB as found in different/diverse plant systems, which include its effect on ABA, cytokinin, and stress tolerance (Tesfahun, 2018). Furthermore, it is hypothesized that environmental stress conditions will reduce SR yield by activating similar mechanisms. Thus, the genes/mechanisms highlighted in the present study can serve for future experiments dealing with the molecular basis of the effect of stress on SR formation.

## Data Availability Statement

All datasets generated for this study are included in the article/**Supplementary Material**.

## Author Contributions

VS and NF conceived and designed the experiments, analyzed the data, and wrote the manuscript. VS performed all the experiments. LS and WL performed gibberellin quantification. HZ helped in anatomical analyses. SS provided technical assistance in part of the experiments and starch analyses. AD-F did bioinformatics analysis. JY and ZP critically discussed the experiments and the results, and reviewed the manuscript. RA helped in interpretation of the anatomical data, vessel formation, and lignification. NF critically reviewed the manuscript. All the authors discussed the results and approved the manuscript.

## Funding

We thank the Chief Scientist of The Ministry of Agriculture and Rural Development, Israel, Grant Agreement No. 20-10-0036 for supporting the research, in the frame of Israel–China collaboration.

## Conflict of Interest

The authors declare that the research was conducted in the absence of any commercial or financial relationships that could be construed as a potential conflict of interest.

## Footnotes

1 http://www.bioinformatics.nl/cgi-bin/primer3plus/primer3plus.cgi

2 https://www.arabidopsis.org

3 https://www.ncbi.nlm.nih.gov

4 http://planttfdb.cbi.pku.edu.cn

5 https://www.genome.jp/tools/motif/

## Abbreviations

AGPase, ADP glucose pyrophosphorylase; AR, adventitious root; *BP, BREVIPEDICELLUS*; CAD, cinnamyl alcohol dehydrogenase; CCoAOMT, caffeoyl-CoA *O*-methyltransferase; C4H, cinnamate 4-hydroxylase; 4CL, 4-coumarate:CoA ligase; GA, gibberellin; GAI, gibberellic acid insensitive; GBSS, granule-bound starch synthase; GID1, GA insensitive dwarf 1; GA2ox, GA 2-oxidase; GA20ox, GA 20-oxidase; GA3ox, GA 3-oxidase; HCT, *p*-Hydroxycinnamoyl-CoA: quinate shikimate *p*-hydroxycinnamoyltransferase; KNOX, Class I knotted 1-like; KO, ent-kaurene oxidase; LR, lateral root; NST/SND, NAC secondary-wall thickening promoting factor/secondary-wall-associated NAC domain protein; PAL, phenylalanine ammonia-lyase; PB, paclobutrazol; PGM, phosphoglucomutase; Ph-HCl, phloroglucinol-HCl; RSA, root system architecture; SP, starch phosphorylase; SR, storage-root; SuSy, sucrose synthase; VND, vascular-related NAC domain; VNI2, VND-INTERACTING2; XND1, XYLEM NAC DOMAIN 1.
