# Supplementary material for: Gibberellin Promotes Sweetpotato Root Vascular Lignification and Reduces Storage-Root Formation
Source: Front Plant Sci. 2019 Nov 15;10:1320. doi: 10.3389/fpls.2019.01320 (PMC6897044; doi:10.3389/fpls.2019.01320)
Supplement: Supplementary file 1 [file DataSheet_1.pdf]

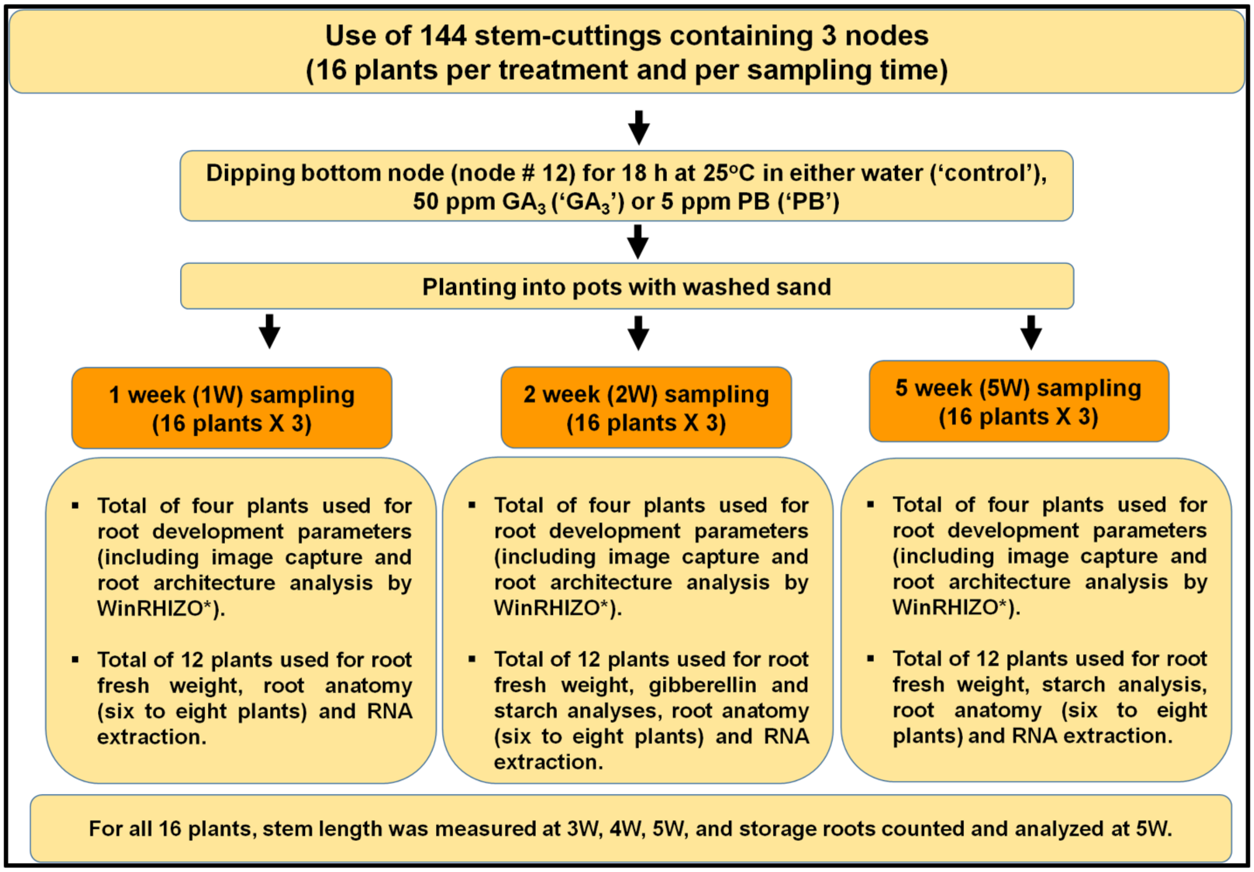

SUPPLEMENTARY FIGURE S1. Schematic representation of the experimental setup. Details are given in Materials and methods section. *Root development parameters included adventitious root number and cumulative length; lateral root number and cumulative length, lateral root density, root volume and root surface area. For anatomy, roots were sampled in FAA. For RNA extraction, roots were immediately frozen at -80°C.

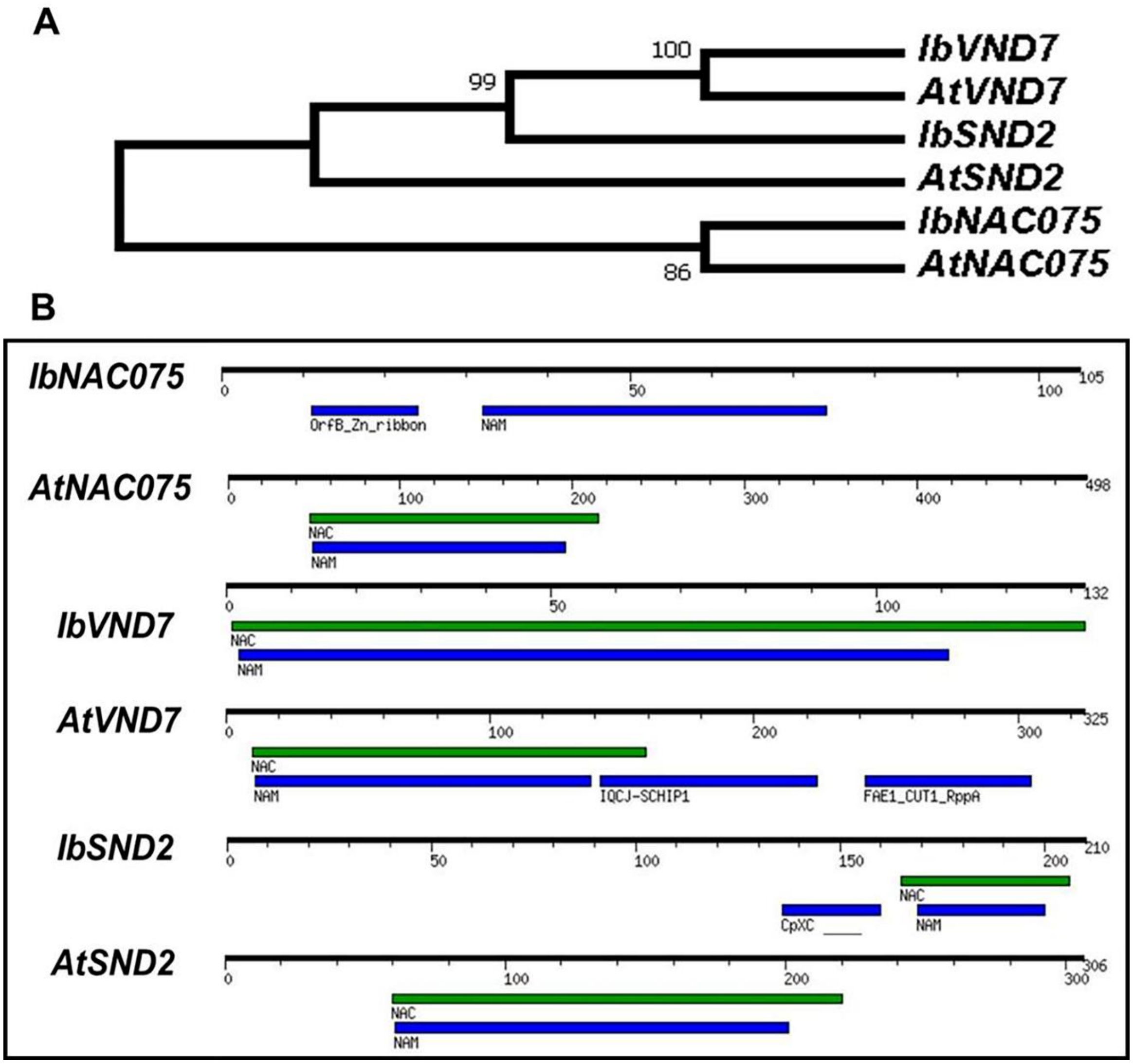

SUPPLEMENTARY FIGURE S2. Identification of potential sweetpotato orthologues of *Arabidopsis* vascular development regulators. Phylogenetic tree showing homology of sweetpotao orthologues/genes to their corresponding *Arabidopsis* genes (A). Phylogenetic tree was constructed using MEGA by neighbor-joining method with 1000 bootstrap replicates. Each gene showing conserved NAC and NAM domains (B). Identification of NAC and NAM domains was done by motif search analysis using MOTIF: Searching Protein Sequence Motifs- GenomeNet (https://www.genome.jp/tools/motif/).

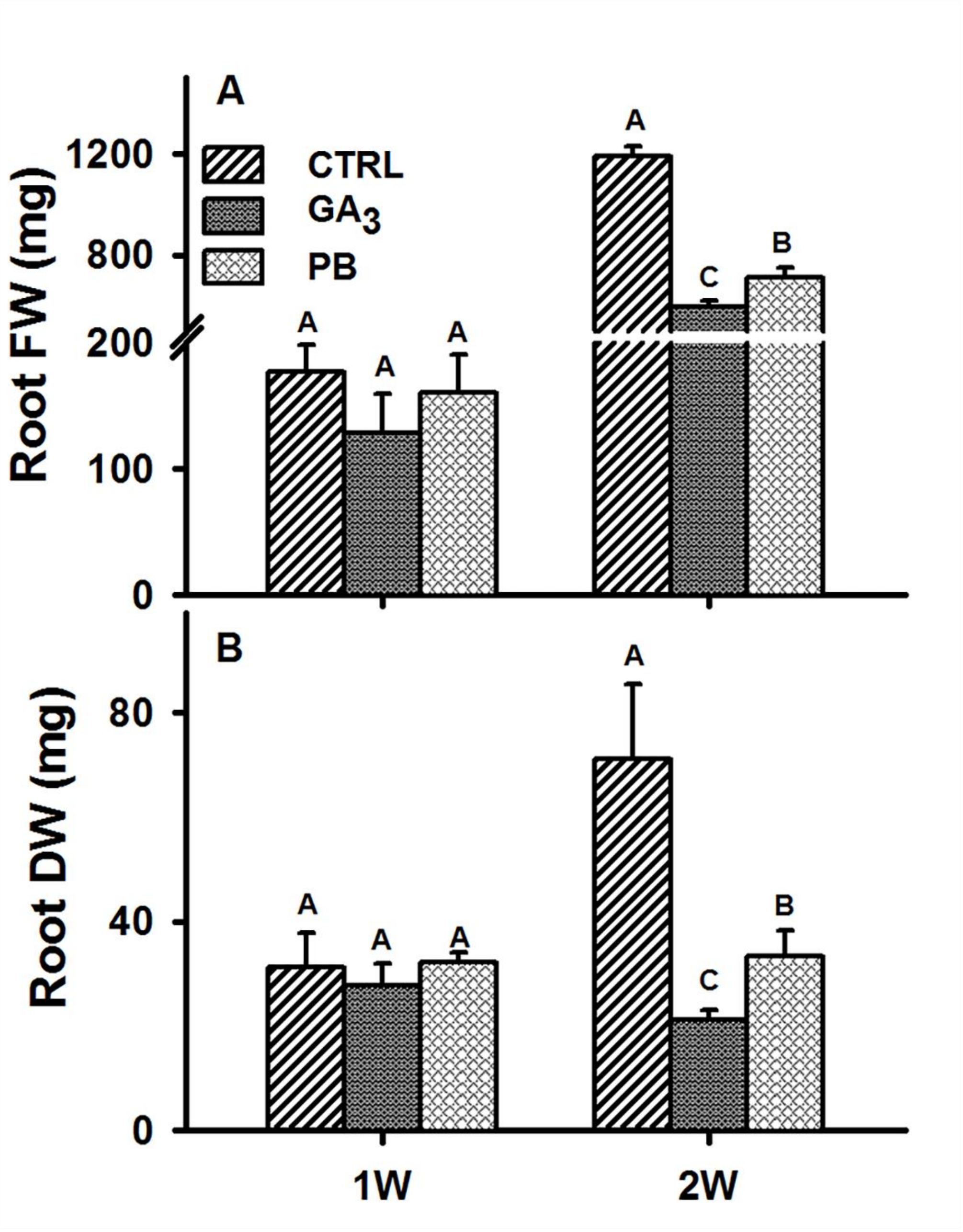

SUPPLEMENTARY FIGURE S3. Effect of GA_3 application on sweetpotato "*Georgia Jet*" root fresh weight (FW; **A**) and dry weight (DW; **B**). Root system weight per plant was recorded at one and two weeks (1W and 2W, respectively) after planting. Bars represent mean of 16 (FW) or 4 (DW) independent biological replicates (plants) ± SE. Significance analysis was performed by using student's t-test (*$P \leq 0.05$)*, where unlike letters represent significant differences between treatments within a sampling group. CTRL, Control (treated with water); GA_3, application of 50 ppm gibberellic acid 3 for two weeks; PB, application of 5 ppm paclobutrazol for two weeks.

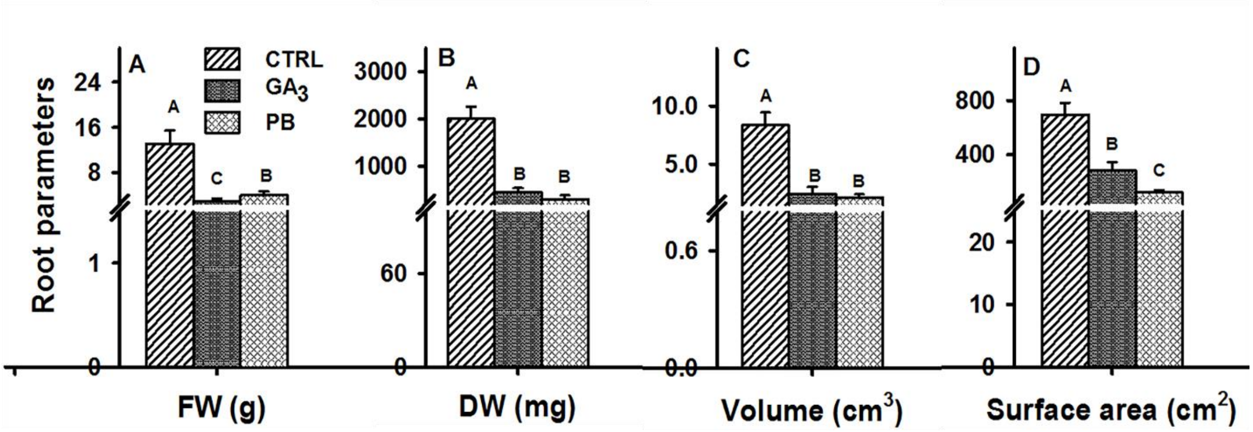

SUPPLEMENTARY FIGURE S4. Effect of GA_3 application on sweetpotato "*Georgia Jet*" root system parameters at the time when storage-root formation was observed. Root system fresh weight (FW; **A**), dry weight (DW; **B**), volume (**C**), and surface area (**D**) per plant were recorded at five weeks after planting. The measured values included all root types present at the time of sampling (adventitious roots; lateral roots and storage-roots). Bars represent mean of four independent biological replicates (plants) ± SE. Significance analysis was performed by using student's t-test (*$P \leq 0.05$)*, where unlike letters represent significant differences between treatments within a sampling group. CTRL, Control (treated with water); GA_3 , application of 50 ppm gibberellic acid 3 for two weeks; PB, application of 5 ppm paclobutrazol for two weeks.

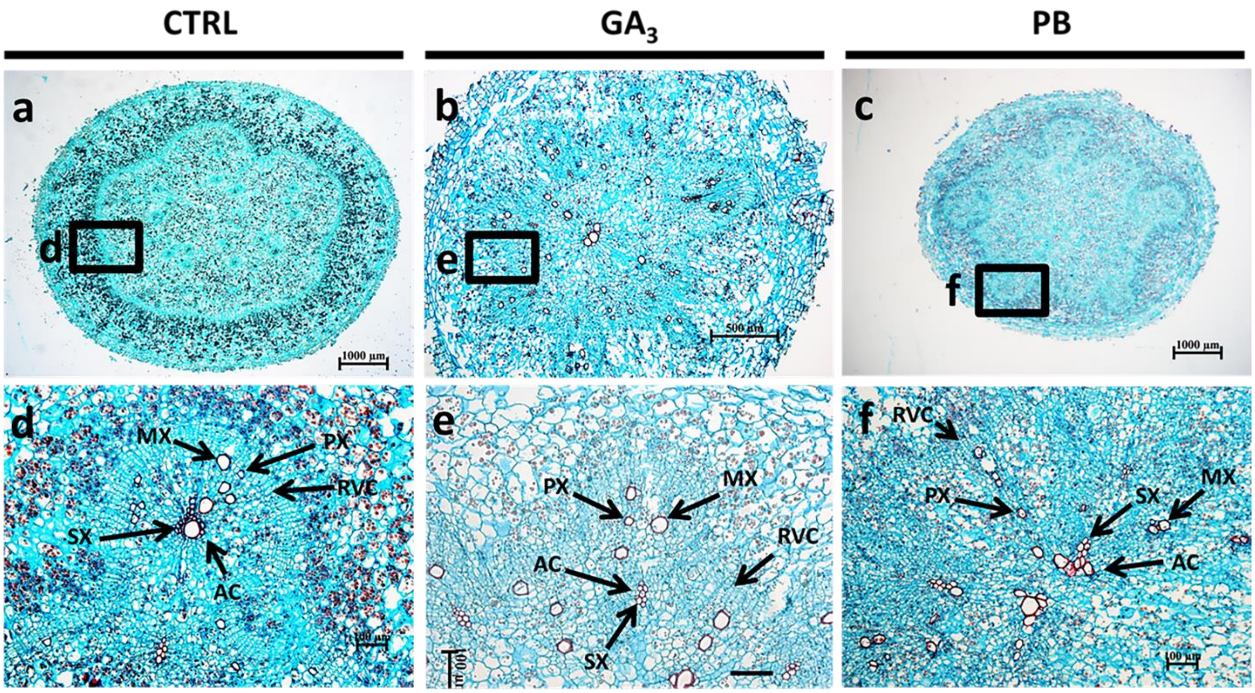

SUPPLEMENTARY FIGURE S5. Effect of GA_3 application on sweetpotato "*Georgia Jet*" storage-root anatomy. Representative cross-sections of SRs sampled at 5 weeks (5W) after planting from control plants (CTRL, treated with water; **a**, **d**), plants treated with 50 ppm gibberellic acid 3 for two weeks (GA_3; **b**, **e**) and plants treated with 5 ppm paclobutrazol for two weeks (PB; **c**, **f**). Sections were stained with safranin and fast green and represent six to eight roots sampled from individual plants. Areas marked by black rectangle (in **a**, **b**, **c**) were enlarged and are presented in **d**, **e** and **f**, respectively. Scale bar = 1000 µm (**a**, **c**), 500 µm (**b**) and 100 µm (**d**, **e**, **f**).
