# Supplementary material for: Gibberellin Promotes Sweetpotato Root Vascular Lignification and Reduces Storage-Root Formation
Source: Front Plant Sci. 2019 Nov 15;10:1320. doi: 10.3389/fpls.2019.01320 (PMC6897044; doi:10.3389/fpls.2019.01320)
Supplement: Supplementary file 2 [file Table_1.pdf]

SUPPLEMENTARY TABLE S1. Primers used for gene expression analysis by qRT-PCR.

***Ipomoea batata* contigs**	***Ipomoea batata* gene name**	**Forward primer**	**Reverse Primer**
S_PBL_c17751	Kurene oxidase (*IbKO*)	GCATAGGAAAAGGCAGTGAGA	CGAGAGCAACCACCAAACC
S_PBL_c36353	Gibberellin 20-dioxygenase (*IbGA2Oox*)	TTTACAGCCCCTTTGTTTGC	GGCCAGCATAATCTTTAACTGTG
S_PBL_c51845	Gibberellin 3-beta-dioxygenase 4 (*IbGA3ox4*)	GAGCCCAACCTAGTGAAACAA	CCAGGACAGGGAGTTGTATAGG
S_PBL_c23111	Gibberellin 2-oxidase1 (*IbGA2ox1*)	AACGTCGGAGATTCTCTTCAGG	TGACAGCCTCGATTTTGCAC
S_PBL_c32	Gibberellin 2-oxidase 3 (*IbGA2ox3*)	CATATCCATAGCTCCCCACAA	CTGGGTGCTAGGGGAGATAGA
S_PBL_c13427	Gibberellin 2-oxidase8 (*IbGA2ox8*)	GTGGGGAAGGCAATATACGA	CGAGGGTATCGATATACACGTAA
S_PBL_c19593	Gibberellin insensitive dwarf1 (IbGID1)	TCCGGCTTAAGGAGGAAGTA	ACATCATGGATTTCCCCAAA
S_PBL_c1873	GA-insensitive (*IbGAI/RGA2*) / DELLA protein	ATTCTCCCCGGTTCACTTG	CGTCGTTCTCCTCCACTCTG
S_PBL_c36855	VASCULAR RELATED NAC-DOMAIN PROTEIN 075 (*IbNAC075*)	AAGAGCATCAGCTGTGTGGA	CAATTCTTGATCTGTTGGGTCA
S_PBL_c32341	VASCULAR RELATED NAC-DOMAIN PROTEIN 7 (*IbVND7*)	TAGAATGGAGCCATGGGACA	AGTTCCCGTCGGATACTTCC
S_PBL_c24252	SECONDARY WALL-ASSOCIATED NAC DOMAIN 2 (*IbSND2*)	GCCTGGTCTACCCGCCG	AGGATGAAGTTTCCGGCTGT
S_PBL_c504	XYLEM NAC DOMAIN1 (*IbXND1*)	ACGCTTTTTACGTCGGAGAG	TGGAAGCGGAATAATCGGAGAG
S_PBL_c4628	VND-INTERACTING2 (*IbVNI2*)	ACCCATACCCATACCCATCTTC	AACTCAACTTCTCCATAACCACC
S_PBL_c17476	VND-INTERACTING2-like (*IbVNI2-like*)	ACCCTAGCTCAGGCTCAGAC	CCCTCGCCAAGAAGTCGTAG
S_PBL_c2312	Phenylalanine ammonia lyase (*IbPAL*)	GGATCCAAGAGTGCAGGTCC	CCTTGTCACATTCCTCCCCG
S_PBL_c7605	Cinnamate 4-hydroxylase (*IbC4H*)	GCGGCAAGAAGTACAAGCTC	CTTGGCGTAATCGGTGAGAT
S_PBL_c18044	4-Coumarate-CoA ligase (*Ib4CL*)	CTGAGGATGAAGTTAAAGAGTTTGTG	GCCTGAGGGAGACTTTGGA
S_PBL_c17752	Hydroxycinnamoyl transferase (*IbHCT*)	CCGTCGCTTACAGCTCCTAC	CGGTGGCTATGTACAGCTTG
S_PBL_c2944	Caffeoyl-CoA-O-methyltransferase (*IbCCoAOMT*)	GAGGCACCCACAAGACTACG	TGGTTGTCTGATTCTCCGCC
S_PBL_lrc53688	Cinnamyl alcohol dehydrogenase (*IbCAD*)	GTCTTGGCGCAGACTCTTTC	TAATGGCACAACAGCGTGAT
S_PBL_c8137	Class I knotted 1-like homeobox (KNOX1) (*IbKN2*)	GCCAGGCAGAAGTTGCTTAG	CAGTGCCGTTTTCTTTGGTT
S_PBL_c31412	Class I knotted 1-like homeobox (KNOX1) (*IbKN3*)	CGCCTAGGTCCATAATCC	TATTTCAAGGCGGTCTCA
S_PBL_c543	Sucrose synthase (*IbSuSy*)	TACTGCATCCTTTCCCAAGC	AGCCATGCTCTCCTTGTCAT
S_PBL_c20112	Phosphoglucomutase (*IbPGM*)	TGAGAAGGATGCTTCCAAGA	GCCAGTGAATTCCTGCATCT
S_PBL_c18129	ADP-glucose pyrophosphorylase alpha subunit (*IbAGPa1*)	CAATTGGAATTGGCAGGAAT	TTTCCCTAGCTGCTTCTTGAAC
S_PBL_c54187	ADP-glucose pyrophosphorylase beta subunit (*IbAGPb1A*)	GATTGGGTTAAAGACGTTGTCATC	CACGAATGGTTGCTTTCTCC
S_PBL_c3042	Granule-bound starch synthase (*IbGBSS*)	GGACGTGCTGAAGGTGATAA	CGAGAGCTCTTGTGACATGC
S_PBL_c1370	Starch phosphorylase (*IbSP*)	GTGTGACACCAAGAAGATGGA	CGCAGTTCTGCCAACTTTTC
JX177360.1	Phospholipase D1a (*IbPLD*)	ATCGGAATCAGCAGTGATGG	ATGATGAGGCAAGCAGTGTG
